# Sequencing of *Pax6* Loci from the Elephant Shark Reveals a Family of *Pax6* Genes in Vertebrate Genomes, Forged by Ancient Duplications and Divergences

**DOI:** 10.1371/journal.pgen.1003177

**Published:** 2013-01-24

**Authors:** Vydianathan Ravi, Shipra Bhatia, Philippe Gautier, Felix Loosli, Boon-Hui Tay, Alice Tay, Emma Murdoch, Pedro Coutinho, Veronica van Heyningen, Sydney Brenner, Byrappa Venkatesh, Dirk A. Kleinjan

**Affiliations:** 1 Institute of Molecular and Cell Biology, Agency for Science Technology and Research (A*STAR), Biopolis, Singapore, Singapore; 2 MRC Human Genetics Unit, MRC Institute of Genetics and Molecular Medicine, University of Edinburgh, Edinburgh, United Kingdom; 3 Institute of Toxicology and Genetics, Karlsruhe Institute of Technology, Eggenstein-Leopoldshafen, Germany; Fred Hutchinson Cancer Research Center, United States of America

## Abstract

*Pax6* is a developmental control gene essential for eye development throughout the animal kingdom. In addition, *Pax6* plays key roles in other parts of the CNS, olfactory system, and pancreas. In mammals a single *Pax6* gene encoding multiple isoforms delivers these pleiotropic functions. Here we provide evidence that the genomes of many other vertebrate species contain multiple *Pax6* loci. We sequenced *Pax6*-containing BACs from the cartilaginous elephant shark (*Callorhinchus milii*) and found two distinct *Pax6* loci. *Pax6.1* is highly similar to mammalian *Pax6*, while *Pax6.2* encodes a paired-less Pax6. Using synteny relationships, we identify homologs of this novel paired-less *Pax6.2* gene in lizard and in frog, as well as in zebrafish and in other teleosts. In zebrafish two full-length *Pax6* duplicates were known previously, originating from the fish-specific genome duplication (FSGD) and expressed in divergent patterns due to paralog-specific loss of *cis*-elements. We show that teleosts other than zebrafish also maintain duplicate full-length *Pax6* loci, but differences in gene and regulatory domain structure suggest that these Pax6 paralogs originate from a more ancient duplication event and are hence renamed as *Pax6.3*. Sequence comparisons between mammalian and elephant shark *Pax6.1* loci highlight the presence of short- and long-range conserved noncoding elements (CNEs). Functional analysis demonstrates the ancient role of long-range enhancers for *Pax6* transcription. We show that the paired-less *Pax6.2* ortholog in zebrafish is expressed specifically in the developing retina. Transgenic analysis of elephant shark and zebrafish *Pax6.2* CNEs with homology to the mouse NRE/Pα internal promoter revealed highly specific retinal expression. Finally, morpholino depletion of zebrafish Pax6.2 resulted in a “small eye” phenotype, supporting a role in retinal development. In summary, our study reveals that the pleiotropic functions of Pax6 in vertebrates are served by a divergent family of *Pax6* genes, forged by ancient duplication events and by independent, lineage-specific gene losses.

## Author Summary

Pax6 is a highly conserved transcription factor with key roles in eye, brain, pancreas, and olfactory system development. In mammals multiple Pax6 isoforms are encoded by a single *Pax6* gene, embedded within a complex regulatory landscape. Here we provide evidence for the presence of multiple *Pax6* loci in other vertebrate species. We show that two *Pax6* genes (*Pax6.1* and *Pax6.2*) are present in the genome of elephant shark (a cartilaginous fish). *Pax6.1* is highly similar to mammalian *Pax6* in terms of structure of the gene locus, protein sequence, and expression pattern; whereas the second gene, *Pax6.2*, codes for a protein lacking the paired domain. We identify orthologs of *Pax6.2* in other vertebrate genomes, such as lizard, *Xenopus*, and teleost fishes, and show it is important for eye development in zebrafish. Additionally, we have characterised a third *Pax6* (*Pax6.3*) present only in some teleost fishes. Phylogenetic analyses indicate that the evolutionary history of the *Pax6* gene family in vertebrates is a result of ancient duplications followed by independent gene losses in different lineages. Sequence comparison of the *cis*-regulatory landscapes around the genes has led to the identification of novel *Pax6* enhancers that provide a link between the diverged *Pax6* family members.

## Introduction

Development is critically dependent on a core set of developmental regulator genes, most of which are highly conserved across metazoans and carry out pleiotropic functions as part of multiple gene regulatory networks. Variation in the functional output from these genes is an important factor in evolutionary divergence between species, yet their essential and pleiotropic role precludes dramatic changes in their primary sequence. Instead it is believed that duplication of key developmental control gene loci, in combination with variation in their spatio-temporal domains and expression levels are major components of this process [1]–[4]. Duplication of gene loci provides an initial freedom from selective pressure to allow divergence between gene loci.

*Pax6* is a developmental control gene with an essential function in the development of eyes throughout the animal kingdom [5], [6]. Its ability to induce the full program for eye formation from ocular and non-ocular imaginal discs in *Drosophila* embryos has revealed it as the first of a small set of master regulators for eye development [7]–[9]. In vertebrates the key role of *Pax6* in eye formation is equally well established. In addition *Pax6* plays important roles in development and maintenance of the endocrine pancreas, the olfactory system and the central nervous system (CNS) where it is required for multiple cellular processes including maintenance of the neuronal progenitor pool at early developmental stages, and neurogenesis at later stages [10]. It is also required for cell migration and axon guidance in parts of the brain (reviewed in [11], [12]). In humans heterozygous disruption of the gene gives rise to the congenital eye malformation aniridia through haploinsufficiency, in some cases accompanied by additional phenotypes such as epilepsy, defective interhemispheric auditory transfer, anosmia or diabetes [13]–[16], while homozygous loss of gene function is incompatible with life [17]. In mice and rats heterozygous mutants have small eyes and exhibit many of the same features as found in aniridia patients [18], [19]. Homozygous mutants die at birth with severe brain malformation and complete lack of eyes and nasal structures [19]. Overexpression of the gene also causes eye malformations [20], [21], indicating that Pax6 dosage is critical for correct eye development.

In mammals a single *Pax6* gene carries out the wide variety of developmental regulatory functions. This is achieved by strict control of its expression through a complex, extended *cis*-regulatory domain containing a large number of tissue-specific enhancers [22]–[29]. While some *cis*-elements are found upstream or within introns of the gene, most of the characterized long-range control elements are found in the downstream region. The importance of distant *cis*-regulatory elements for *Pax6* gene expression has been highlighted by the existence of aniridia patients with chromosomal abnormalities (deletions/translocations) that separate these elements from the body of the gene [30]–[32]. Investigation of the locus beyond the patient breakpoints by DNaseI hypersensitivity mapping led to the identification of a region containing several *cis*-regulatory elements, embedded within the introns of an adjacent gene, *Elp4*, forcing synteny conservation between the genes [25], [33], [34].

In addition to a large array of distal enhancers, the single mammalian *Pax6* gene uses at least three different promoters, P0, P1 and Pα, and the resulting transcripts produce three protein isoforms, Pax6, Pax6(+5a) and Pax6ΔPD [35]–[37]. The canonical *Pax6* isoform encodes a paired and homeodomain containing transcription factor with a PST-rich transactivation domain at the C-terminus. Inclusion of an alternative exon, exon 5a, results in a protein with an interrupted paired domain that recognizes a different DNA binding sequence [35]. The paired-less ΔPD isoform is produced from a transcript initiating at an internal promoter Pα located in intron 4 of the gene [27]. The resulting protein contains the paired-type homeodomain and transactivation domain, but lacks the N-terminal paired domain. The function of this isoform is unknown, but overexpression has been shown to cause a microphthalmia phenotype in mice [24], [38], [36]. Apart from its DNA-binding capacity the homeodomain is also suggested to function in protein-protein interactions and could be involved in dimerisation [39].

In zebrafish the role of *Pax6* is fulfilled by duplicate *Pax6* genes, *Pax6a* and *Pax6b*, thought to have arisen by the fish-specific whole genome duplication event (FSGD). The FSGD is estimated to have taken place around 320 million years ago in the teleost fish lineage [40], and is often cited as the main contributing factor in the emergence of the large diversity of teleost fish, which make up nearly half of all vertebrate species [41]–[43]. The FSGD is a third genome wide duplication event (3R) that follows two earlier rounds (1R, 2R) of whole genome duplications (WGD) that occurred very early in the evolution of vertebrates [44], though debate about the nature and timing of these WGD events still continues.

As proposed nearly 40 years ago, genome duplication events are powerful drivers of evolution [45]. Genome or gene duplication events provide an initial freedom from selective pressure, and thus create an opportunity for modification or mutation of gene duplicates, as well as for alteration of the *cis*-regulatory landscape around the duplicate gene loci, while critical functions are maintained by the other copy under selective pressure. The various ways that can lead to retention of both gene copies following a duplication event are explained by the Duplication-Degeneration-Complementation (DDC) model [46]. Commonly, particularly in genes with a single function, one of the duplicates will start to accumulate mutations and degenerate beyond recognition over time, leaving the other copy to fulfill its single function (non-functionalization) [47]; Retention of both duplicates is more likely in pleiotropic genes, a category that includes many developmental regulatory genes. A number of scenarios can lead to such an outcome: one of the duplicates may acquire a novel function (neo-functionalization), the functions of the ancestral gene may become divided between the duplicates (sub-functionalization), or a combination of these (neo-subfunctionalization) [46]–[48]. The relative contribution of each of these possibilities is likely to be different for each gene locus and between species.

In many cases the functional divergence of gene duplicates is driven by changes in their *cis*-regulatory domains. In recent years identification of *cis*-regulatory elements has been greatly facilitated by comparative analysis of sequence conservation between distantly related species [49]. Compared to surrounding neutral sequences, sequences with important regulatory function are maintained under selective pressure and stand out as conserved non-coding elements (CNEs). However, functionally conserved regulatory regions with little or no sequence conservation have also been identified [28], [50], while sequence-similar enhancers can drive dissimilar expression patterns [51], [52] fuelling the debate on whether sequence conservation is necessary for functional conservation.

It is now thought that a massive appearance of novel CNEs has occurred early in the evolution of jawed vertebrates [53]. Comparisons with available sequence from the jawless vertebrate lamprey suggests it contains far fewer CNEs that are also shorter and less well conserved, while very few conserved non-coding elements can be identified in amphioxus [54], or ascidians (*Ciona*). In addition to the large-scale acquisition of ancient enhancers during the early gnathostome period [55], additional novel CNEs have been recruited at later stages in the evolution of specific lineages [56]. On the other hand, some ancient CNEs have been lost independently in different bony vertebrate lineages [57]. Thus absence of sequence conservation in teleosts at the position of a conserved element in the tetrapod locus could either indicate the loss of that element in fish species, or gain of the element in the tetrapod lineage.

Two *Pax6* genes were previously identified in the zebrafish genome [58], and comparative analysis of their genomic loci suggested their evolution has largely followed the DDC model of divergence and complementation [59]. This led us to ask whether duplicate copies of *Pax6* could also be identified in other teleost fish species and how the duplicate copies may have diverged in those species. Here we show that duplicate *Pax6* genes are present in several other teleost species such as medaka, stickleback, fugu and Tetraodon (acanthopterygians). Examining the patterns of non-coding sequence conservation around the duplicate loci reveals a strong difference in the evolutionary divergence of the *Pax6* loci in the acanthopterygians when compared to the zebrafish *Pax6a*/*b* divergence. While in zebrafish both loci have retained a large and overlapping portion of the *cis*-regulatory repertoire, the difference in the *cis*-regulatory domains of the duplicates in other teleosts is much more dramatic. Comparison with the mammalian locus indicates conservation of the ancient *cis*-regulatory landscape at the *Pax6a* loci, and a complete absence of conserved *cis*-elements at the *Pax6b* loci. In combination with a loss of the potential to encode the alternative exon5a this suggested a different evolutionary origin for the *Pax6b* loci in the acanthopterygians.

To relate our observations to a more ancestrally diverged species, we proceeded to obtain genomic sequence for the *Pax6* locus of the elephant shark (*Callorhinchus milii*), a cartilaginous fish. Cartilaginous fishes are the most basal group of living jawed vertebrates and hence constitute a critical outgroup for bony vertebrates. The elephant shark is particularly attractive for comparative studies into the evolution of *cis*-regulatory landscapes in different jawed vertebrate lineages. Initial low coverage (∼1.4×) sequencing of its relatively small genome (910 Mb) revealed a greater complement of conserved non-coding sequences between the elephant shark and human genome than between human and zebrafish [60]. Surprisingly, screening of our elephant shark BAC library indicated the existence of a second *Pax6* locus in this species. Sequence analysis of the BAC containing this locus revealed that this gene is a paired-less *Pax6* gene, which we named *Pax6.2*. To determine whether the existence of this gene indicates a shark-specific duplication, or is the result of an ancient duplication event predating the split between cartilaginous fishes and bony vertebrates, we searched the genomes of other species for the presence of additional *Pax6* genes using synteny relations from the elephant shark *Pax6.2* locus. This approach revealed the presence of *Pax6.2* orthologs in *Xenopus*, lizard, zebrafish and other teleosts. Thus the zebrafish genome contains 3 *Pax6* genes. Further evidence for the shared ontogeny of these orthologs comes from identification and analysis of conserved *cis*-elements located near the genes. We show that these elements share both sequence and functional conservation, and the study reveals them as ancient enhancers linked to *Pax6* regulation from the earliest stages of vertebrate evolution. Based on our phylogenetic analyses we present a model for the evolutionary history of the *Pax6* gene family in vertebrates. In this model the two rounds of WGD at the base of the vertebrate lineage gave rise to four *Pax6* genes. One of these was lost early on, while a variable subset of the remaining three has been retained in different species as a result of independent divergences and gene losses in different lineages. In the teleost lineage the FSGD produced further duplicates of the three *Pax6* genes of which variable subsets remain in contemporary species.

## Results

### Different origins of the *Pax6* duplicates of zebrafish versus acanthopterygian teleosts

**Figure 1:**
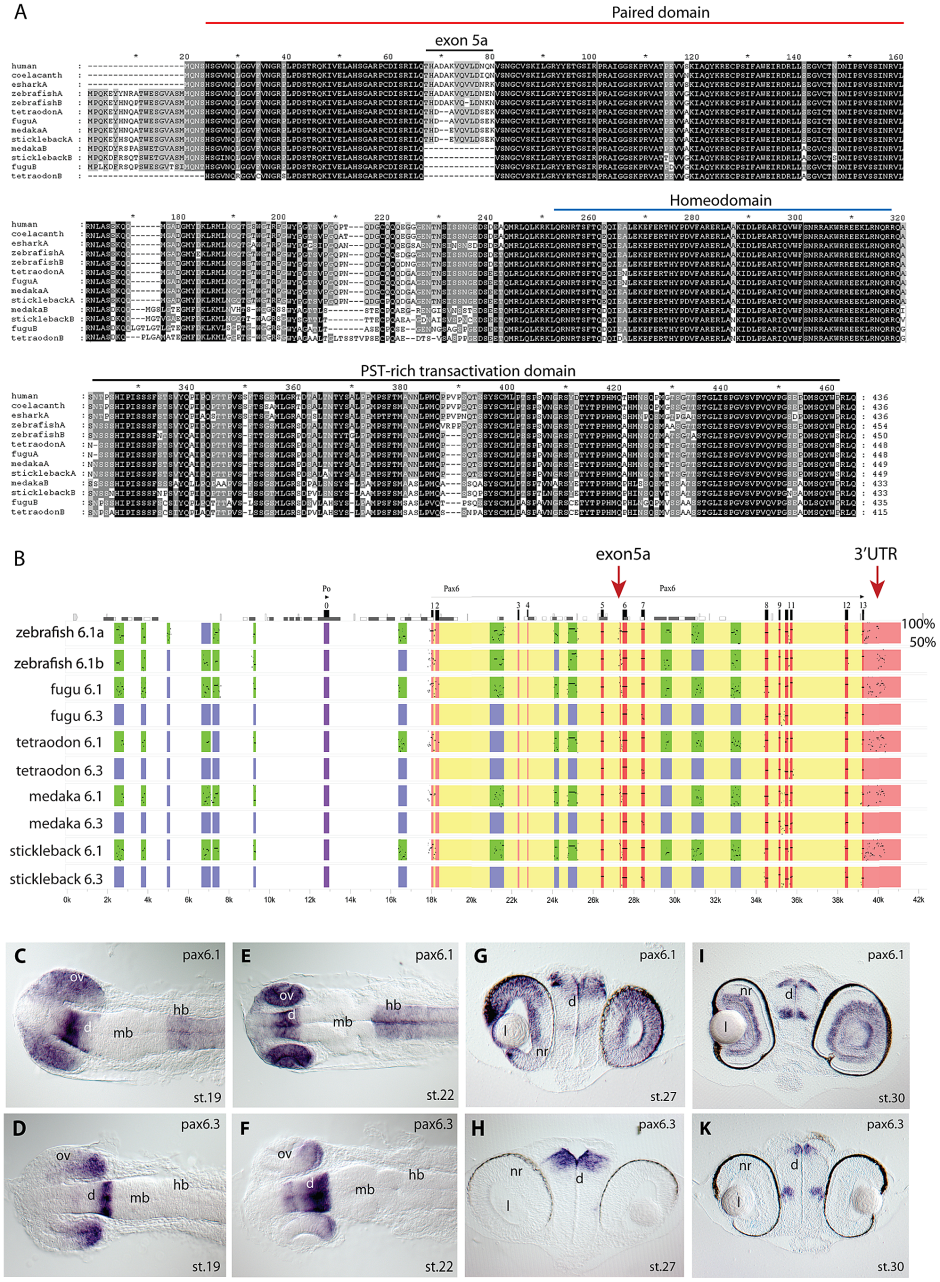
Pax6 gene duplicates in teleost fish. A) Alignment of Pax6 proteins. Amino acid sequences of duplicate Pax6 proteins from various fish species are aligned along those from a variety of tetrapods and elephant shark. Pax6 proteins are highly conserved across vertebrates, particularly in the paired, homeo- and transactivation domains. The potential to encode an N-terminal protein extension is present in all fish *Pax6* genes. The alternative exon 5a is present in the *Pax6* genes of tetrapods, in one of the *Pax6* duplicates in fish, as well as in the second zebrafish *Pax6* gene. The other *Pax6* genes in the acanthopterygian teleosts lack the alternative exon 5a. B) Percentage Identity Plot (PIP) showing multispecies sequence comparison of the genomic region around the *Pax6* transcription unit using human *PAX6* locus as baseline sequence. The plot highlights a number of features that indicate different evolutionary origins of the duplicated *Pax6* genomic loci in fish species. While strong conservation of exonic sequences (red boxes indicate their positions, black lines/dots show the level of conservation) is seen for the duplicate genes in all fish species, a conspicuous absence of conserved elements is observed in the upstream and intronic regions of the second *Pax6* loci of medaka, stickleback, fugu and *Tetraodon*. In contrast, in zebrafish both duplicate loci contain a largely overlapping array of conserved non-coding elements (CNEs). CNEs are highlighted by green boxes, while their absence is shown by blue boxes. Exon 5a, an alternatively spliced exon located immediately upstream of exon 6 (red arrow), is present in both zebrafish *Pax6* loci (*Pax6.1a* and *b*), but absent from the second *Pax6* loci (*Pax6.3*) of the other teleosts (note the absence of black dots/lines in the red box for the exon5a position). Similarly, the gene has conserved sequences in its 3′UTR (red arrow) that are present in all canonical *Pax6* (*Pax6.1*) loci, and are subpartitioned between the zebrafish *Pax6* duplicates, but are not found in the 3'UTRs of the *Pax6.3* gene of other fish species. C–K) RNA *in situ* analysis of medaka *Pax6* genes during early embryonic stages: C) At stage 19 expression of medaka *Pax6.1* is seen in the optic vesicle (ov), diencephalon (d) and hindbrain (hb), but is absent from the midbrain (mb). D) Expression of *Pax6.3* at the same stage is seen in the distal part of the optic vesicle and in the posterior diencephalon. E) At stage 22 *Pax6.1* expression is seen in the optic cup (oc), diencephalon and hindbrain, while F) *Pax6.3* signal is maintained in the posterior half of the optic cup and has increased in the diencephalon. G) A cross section at stage 27 shows *Pax6.1* expression in the neuroretina (nr) and lens (l) epithelium of the eye, and in the dorsal diencephalon. H) In contrast *Pax6.3* signal is no longer seen in the retina, but strong expression is maintained in the dorsal diencephalon. I) By stage 30 medaka *Pax6.1* expression is restricted to the lens epithelium, the ganglion cell layer and the inner nuclear layer in the retina, and in the dorsal and medial diencephalon and ventral nerve tracts. K) *Pax6.3* expression is limited to the dorsal and medial diencephalon.

To identify duplicate *Pax6* genes in teleost fishes other than zebrafish, we searched the genomes of four other teleost fish species for which genomic sequences were available in the Ensembl database: medaka (*Oryzias latipes*), stickleback (*Gasterosteus aculeatus*), green spotted pufferfish (*Tetraodon nigroviridis*) and fugu (*Takifugu rubripes*). We found evidence for the presence of duplicated *Pax6* gene loci in these four species of acanthopterygians. We compared the genes from these five teleost fish species with the *Pax6* gene from a selection of other vertebrate species. Alignment of the amino acid sequences of the encoded proteins demonstrates a high level of similarity between the duplicated fish proteins and tetrapod Pax6 in all protein domains, including the paired domain, homeodomain and PST-rich transactivating regions (Figure 1A). The two major isoforms of full length Pax6 in tetrapods, Pax6 and Pax6(+5a), differ by a 14 amino acid insertion into the paired domain, encoded by an alternative exon 5a [35]. This alternative exon is present in the *Pax6a* gene of all five teleost fishes, and it is also found in the zebrafish *Pax6b* gene. Comparisons of the alternative exon between human and the fish species indicate significant variation between the peptide encoded by the exon 5a, both with respect to the amino acid sequence and length of the peptide (Figure 1A). However, while exon 5a is found in both zebrafish *Pax6* duplicates, the exon is notably absent from the second *Pax6* gene in the other four teleost species (Figure 1A). The region between exons 5 and 6 of the *Pax6b* genes in these fishes lacks any sequence homology to the exon, and manual searching of translated sequence in all reading frames failed to detect evidence for the presence of this exon. This indicates that the *Pax6b* genes of medaka, stickleback, fugu and *Tetraodon* do not have the ability to encode a Pax6(+5a) isoform, while both zebrafish *Pax6a* and *Pax6b* have retained this ability.

In tetrapods translation of full length *Pax6* strictly starts at the ATG initiator codon located in exon 4. It has been noted that in teleost fish translation can also start in exon 2, creating an N-terminal extension to the encoded Pax6 protein [37], [58], [61]. Alignment of available ESTs and Genewise protein predictions shows most teleost *Pax6a* and *Pax6b* genes have the ability to code for this N-terminal protein extension, suggesting this teleost specific feature was acquired early in evolution and has been retained throughout the divergence of teleost *Pax6* genes (Figure 1A).

We next downloaded the sequence scaffolds around the genes, and performed sequence alignments using PipMaker [62] using the human genomic region as reference sequence, to examine divergence of the paralogous genomic loci in the teleost species (Figure 1B). Examining the patterns of sequence conservation around the gene duplicates reveals a much more dramatic divergence between the paralogous loci in the four teleost fish, when compared to the relatively balanced sub-partitioning of conserved elements around the *Pax6* duplicates in zebrafish. While in zebrafish both loci have retained a large and overlapping portion of the *cis*-regulatory repertoire, the divergence in the other teleosts has led to conservation of most of the ancient *cis*-regulatory landscape only at the *Pax6a* loci. The acanthopterygian *Pax6b* loci on the other hand have experienced a dramatic divergence or loss of *cis*-elements, such that, with one exception (see below), no recognizably conserved non-coding sequences remain in the locus in comparison with the mammalian or zebrafish *Pax6* loci (Figure 1B). Furthermore, the blocks of sequence conservation in the 3′UTR are also absent from the acanthopterygian *Pax6b*, while found sub-partitioned between the zebrafish *Pax6a* and *Pax6b* loci. The lack of alternative exon 5a, the absence of CNEs and the differences in synteny conservation around the genes (see below) strongly suggest that the *Pax6* duplicates of medaka, stickleback, fugu and *Tetraodon* originate from a separate duplication event compared to the zebrafish duplicates. To distinguish between these separate sets of *Pax6* genes we will refer to the canonical *Pax6* gene as *Pax6.1* in species with multiple *Pax6* loci. Thus all fish *Pax6a* genes will be termed *Pax6.1*, while the duplicate zebrafish *Pax6a* and *Pax6b* genes will be redefined as *Pax6.1a* and *Pax6.1b*. The second full-length *Pax6* gene found in acanthopterygians will hereafter be referred to as *Pax6.3*.

### The early expression pattern of medaka *Pax6.3*

As the near complete lack of CNEs between the *Pax6.1* and *Pax6.3* loci could suggest non-functionalization of the acanthopterygian *Pax6.3* genes, we carefully inspected the coding sequences of the genes. All *Pax6.3* genes contain open reading frames that encode clear Pax6 homologs. The conservation of ORFs in all four acanthopterygian species examined suggests that the gene is functional, which is further supported by the existence of ESTs for the *Pax6.3* gene in medaka (GenBank BJ013007 and AM298948). To look for the expression pattern of *Pax6.3*, a probe was made from one of the medaka ESTs (clone MF01SSA182E11) and used for RNA *in situ* hybridizations in early medaka embryos (Figure 1C–1K). At stage19, weak expression of *Pax6.3* was observed in the anterior diencephalon, whereas the posterior diencephalon and posterior part of the optic vesicle showed stronger expression (Figure 1D). Broader expression was seen for medaka *Pax6.1* in the diencephalon, hindbrain and entire optic vesicle at this stage (Figure 1C, and [63]). At stage 22, the *Pax6.3* expression domain in the diencephalon appears widened and persists in the posterior optic cup (Figure 1F), while *Pax6.1* expression is maintained throughout the optic cup, diencephalon and hindbrain (Figure 1E). Expression of *Pax6.3* persists in the diencephalon at stage 27 and 30, but eye expression was no longer observed (Figure 1H, 1K). In contrast *Pax6.1* expression is maintained in the diencephalon, hindbrain and eyes at stage 27 (Figure 1G), and is restricted to the ganglion cell layer and inner nuclear layer of the neural retina at stage 30 (Figure 1I). This restricted expression pattern of medaka *Pax6.3* mRNA in combination with the presence of an intact ORF suggests that the gene is functional, and is subject to regulated control of expression. We therefore generated a sequence alignment of the teleost *Pax6* gene loci, using the stickleback *Pax6.3* locus as baseline sequence (Figure S1). The alignment indicated the presence of a small number of CNEs that are conserved specifically between the *Pax6.3* loci. As expected no non-coding conservation was found with the *Pax6.1* loci, with exception of one small region of conserved sequence (E-200, see below).

### Comparison of cis-regulatory activity in intron 7 of *Pax6*

**Figure 2:**
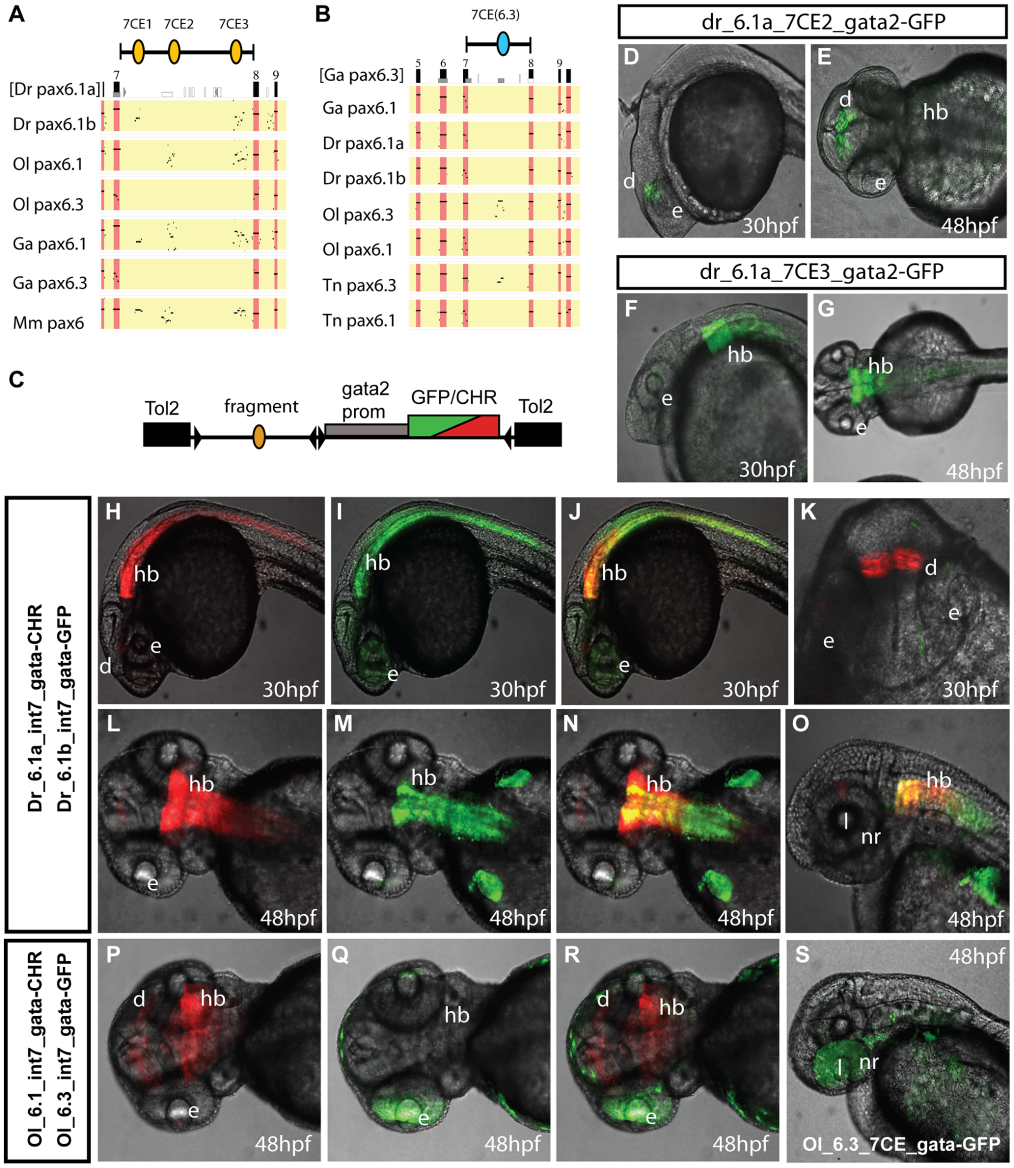
Comparison of functional activity driven by zebrafish and medaka intron 7 sequences. A, B) Comparison of full *Pax6* intron 7 sequences of multiple teleost species visualized as PIP plots. A) PIP plot with zebrafish *Pax6.1a* intron 7 as baseline sequence against intron 7 sequences of the *Pax6.1* and *Pax6.3* genes of zebrafish (*Danio rerio* (Dr)), medaka (Oryzias latipes (Ol)), stickleback (*Gasteroteus aculeatus* (Ga)), tetraodon (Tetraodon nigroviridis (Tn)) and mouse (*Mus musculus* (Mm)), showing lack of sequence conservation of *Pax6.1* intron 7 with the *Pax6.3* loci. A variable subset of CNEs has been conserved in the *Pax6.1* loci. B) PIP plot using stickleback intron 7 as base sequence against the intron 7 regions of the *Pax6.1* and *Pax6.3* loci from multiple teleosts, showing the presence of a conserved element unique to the *Pax6.3* genes only. C) To assess whether patterns of sequence conservation are reflected at the functional *cis*-regulatory level the full intron 7 sequences from zebrafish *Pax6.1a* (Dr6.1a_int7), zebrafish *Pax6.1b* (Dr6.1b_int7), medaka *Pax6.1* (Ol6.1_int7) and medaka *Pax6.3* (Ol6.3_int7) were cloned in front of a gata2 minimal promoter-reporter cassette in a Tol2-2way reporter vector system. Most fragments were cloned with both GFP and mCherry as fluorescent reporter to allow combinatorial analysis in dual fluorescence reporter transgenic zebrafish. (D–G) In addition smaller fragments containing the individual 7CE1, 7CE2 and 7CE3 elements from the zebrafish *Pax6.1a* gene, and the 7CE(6.3) element from the medaka *Pax6.3* gene were also cloned and used to produce transient transgenic zebrafish. (D, E) Lateral and dorsal views of transgenic fish for the Dr6.1a_7CE2 element show expression in the diencephalon (d) at 30 and 48 hours post fertilization (hpf). (F, G) Lateral and dorsal views of transgenic fish for the 7CE3 element of zebrafish *Pax6.1a* show expression in the hindbrain (hb) at 30 and 48 hpf. (H–O) Expression driven by the full intron 7 sequences of zebrafish *Pax6.1a* and *Pax6.1b* is consistent with the presence or absence of the 7CE2 and 7CE3 elements. mCherry fluorescence at 30 and 48 hpf recapitulates the combined pattern of the 7CE2 and 7CE3 elements with expression seen in hindbrain (hb) and diencephalon (d). GFP fluorescence, driven by zebrafish *Pax6.1b* intron 7 is observed in the hindbrain and neural tube of transgenic fish, but no signal is seen in the diencephalon in accordance with the absence of the 7CE2 element from the Dr6.1b intron7. A difference in the detail of hindbrain/neural tube expression driven by the Dr6.1a and 6.1b intron 7 sequences is also observed with a stronger and wider expression of Dr6.1a_int7 in the hindbrain and decreasing towards the caudal neural tube, while Dr6.1b_int7 driven expression is narrower in the hindbrain but maintained more evenly along the neural tube. l, lens; nr, neuroretina. (P–S) Medaka *Pax6.1* intron 7 drives expression in a similar pattern to zebrafish *Pax6.1a* with clear expression in diencephalon and hindbrain, with decreasing levels in the neural tube, in accordance with the conservation of the 7CE2 and 7CE3 elements in the intron. Medaka *Pax6.3* intron 7 (Ol6.3_int7) drives GFP reporter expression in the eye (e) of transgenic zebrafish, and this expression is replicated when the 7CE(6.3) element, conserved only in *Pax6.3* loci, is used on its own.

To assess how the presence or absence of particular CNEs correlates with functional *cis*-regulatory activity we focused on intron 7 of the *Pax6* gene. We have previously studied the murine intron 7 in transgenic mice and shown it to contain a number of tetrapod conserved enhancers [23]. These elements show a variable pattern of conservation in the fish loci (Figure 2A). As it contains a divergent complement of CNEs and, importantly, is flanked by the clearly recognizable landmarks of exons 7 and 8 to demarcate the region, we considered intron 7 particularly well-suited to study the correlation between sequence conservation patterns and functional enhancer activity. We cloned the full intron 7 sequences from the zebrafish *Pax6.1a* and *Pax6.1b* and medaka *Pax6.1* and *Pax6.3* genes and made reporter constructs for analysis in transgenic zebrafish. We also made reporter constructs for individual intron 7 CNEs from the zebrafish *Pax6.1a* locus to assess their contributions to the full intron 7 expression pattern. Analysis of the individual 7CE1, 2 and 3 elements indicated that expression in the diencephalon is contributed by the 7CE2 element (Figure 2D, 2E) and expression in the hindbrain by 7CE3 (Figure 2F, 2G). Reporter analysis of the 7CE1 element did not reveal any consistent expression pattern in transient transgenic fish. The 7CE2 and 7CE3 expression patterns in diencephalon and hindbrain are in agreement with the expression sites observed with the murine elements in reporter transgenic mice. However, we found no evidence for 7CE1 driven expression in the eye, or for consistent ectopic activity in the heart for 7CE2 as found in transgenic mice previously [23]. Next we examined reporter expression driven by the full intron 7 sequences from the *Pax6* gene duplicates of zebrafish and medaka. We found that zebrafish *Pax6.1a* intron 7 (Dr6.1a-int7) drives strong reporter expression in the hindbrain and diencephalon of transgenic fish, but again no consistent expression was observed in the eyes (Figure 2H, 2J, 2K, 2L, 2N, 2O). Reporter expression driven by *Pax6.1b* full intron 7 (Dr6.1b-int7) is seen in the hindbrain only (Figure 2I, 2J, 2K, 2M, 2N, 2O). The fluorescence in the fin buds seen in Figure 2M, 2N, 2O is due to the site of integration and was not observed in other transgenic fish. The use of a dual fluorescence reporter system allows direct comparison of the expression patterns of two constructs in the same transgenic fish. Close examination of the reporter fluorescence patterns in the hindbrain of zebrafish transgenic for both the *Pax6.1a* full intron 7 and *Pax6.1b* full intron 7 constructs indicates that while the intron 7 sequences from the zebrafish *Pax6.1a* and *Pax6.1b* loci both drive expression in the hindbrain, the patterns are not fully overlapping. The zebrafish *Pax6.1a* region drives strong expression throughout the width of the hindbrain neural tube, while expression driven by the *Pax6.1b* region is more strongly concentrated along the midline. Furthermore, *Pax6.1b* intron 7 driven expression extends along the length of the neural tube from the hindbrain towards the caudal end, while *Pax6.1a* intron 7 driven expression is strong in the hindbrain segment but diminishes in level more caudally (Figure 2J, 2N, 2O).

Alignment of the full zebrafish and medaka intron 7 sequences indicates conservation of the 7CE2 and 7CE3 elements in the medaka *Pax6.1* locus and a lack of conservation in the medaka *Pax6.3* locus (Figure 2A). We analyzed reporter expression patterns for the medaka intron 7 constructs from both loci in transgenic zebrafish. Reporter fluorescence driven by medaka *Pax6.1* intron 7 (Ol6.1-int7) was seen in hindbrain and diencephalon in a pattern that closely overlapped with zebrafish *Pax6.1* intron 7 (Figure 2P, 2R). Next we assayed reporter expression driven by medaka *Pax6.3* intron 7. Surprisingly, while having no conservation to the intron 7 sequences of the tetrapod or teleost *Pax6.1* genes, Ol6.3-int7 consistently elicited reporter expression in the eyes of transgenic zebrafish. The retinal expression driven by Ol6.3-int7 correlates with the *Pax6.3* RNA *in situ* signal in the eye of early medaka embryos, though it extends more anteriorly as well (Figure 2Q, 2R, 2S). In later stage embryos reporter fluorescence was also seen in the lens. As sequence alignment had shown the presence of a *Pax6.3* loci specific conserved element in intron 7 (Figure 2B), we made transgenic zebrafish with the medaka *Pax6.3* 7CE element on its own, and again observed reporter expression in the eye (Figure 2S), suggesting this *Pax6.3* locus-specific CNE drives *Pax6.3* expression in the eye.

### The elephant shark genome contains two *Pax6* loci

**Figure 3:**
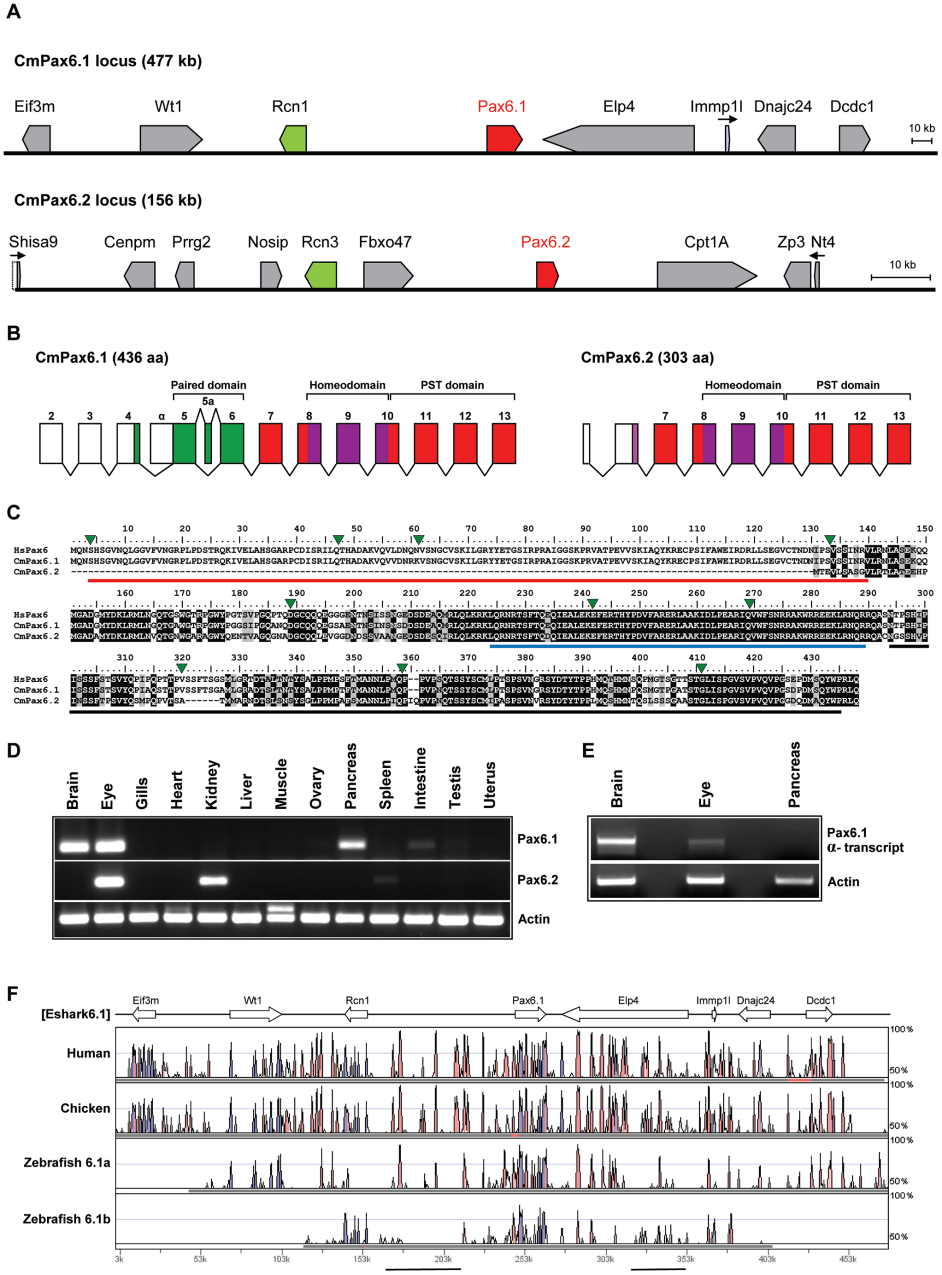
The elephant shark genome contains two *Pax6* genes. A) Schematic representation of genes found in the two elephant shark *Pax6* loci. Genomic sequences for the loci were obtained by complete sequencing of BACs making up the contigs depicted. The elephant shark *Pax6.1* locus (CmPax6.1) is identical in gene content and order to the syntenic region around mammalian *Pax6*. The elephant shark *Pax6.2* (CmPax6.2) gene is found in a different synteny region, which does not have any gene in common with the *Pax6.1* locus other than a paralog of reticulocalbin 3 gene (*Rcn3*) upstream of *Pax6.2*. *Eif3m*. eukaryotic translation initiation factor 3, subunit M; *Rcn1*, reticulocalbin 1; *WT1*, Wilms tumor 1; *Elp4*, elongation protein 4; *Immp1l*, inner mitochondrial membrane peptidase 1 like; *DnaJC24*, DnaJ, subfamily C member 24; *Dcdc1*, doublecortin domain containing 1; *Shisa9*, Shisa homolog 9; *Nosip*, nitric oxide synthase interacting protein; *Rcn3*, reticulocalbin 3, *Prrg2*, proline rich G-carboxyglutamic acid 2; *Fbxo47*. F-box protein 47; Cpt1A, carnitine palmitoyltransferase 1A; Zp3, zona pellucida glycoprotein 3; *Ntf4*, neurotrophin 4. B) Gene structures of the elephant shark *Pax6.1* and *Pax6.2* genes. Exon structure of elephant shark *Pax6.1* is identical to the human *PAX6* gene. In comparison, elephant shark *Pax6.2* lacks the N-terminal exons and encodes a paired-less Pax6 protein. Exon numbering follows that of *Pax6.1*. Paired domain encoding exons are shown in green, homeodomain containing exons in purple, and other coding parts of the gene, including the PST (proline, serine, threonine rich) domain are shown in red. C) Sequence alignment of the elephant shark Pax6.1, Pax6.2 and human PAX6 proteins highlights the absence of the paired domain in elephant shark Pax6.2 (red line) and the high level of conservation in the homeobox (blue line) and PST domain (black line). D) The elephant shark *Pax6.1* and *Pax6.2* genes have tissue-specific expression patterns. rtPCR analysis in 13 different adult elephant shark tissues revealed expression of *Pax6.1* in the brain, eye and pancreas, with a weaker band in intestine. Elephant shark *Pax6.2* on the other hand is expressed in the eye and kidney. E) In mammals a paired-less Pax6 isoform is produced from an internal Pα promoter. A similar transcript is also produced from the elephant shark *Pax6.1* gene in the brain and at lower level in the eye, but not in pancreas. F) VISTA plot of the SLAGAN alignment between the elephant shark *Pax6.1* locus and the human, chicken and zebrafish *Pax6* loci demonstrates a high degree of non-coding sequence conservation between the elephant shark, human and chicken. Conservation of long-range elements (black underlined regions) is much lower in the zebrafish loci. CNEs were predicted at a cut-off of ≥70% identity across >100 bp windows. Purple peaks represent conserved exons and pink peaks conserved noncoding elements.

**Figure 7:**
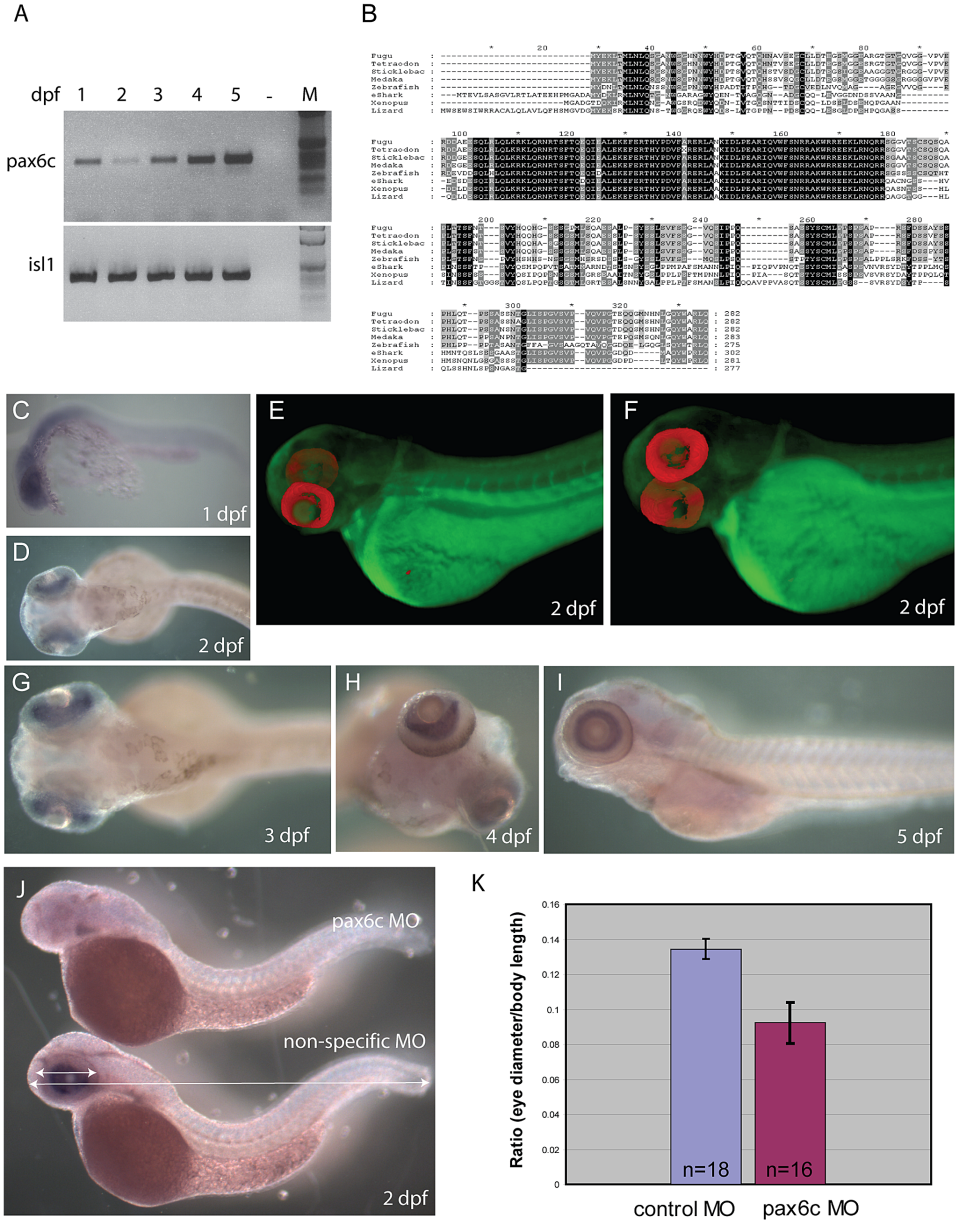
Characterization of zebrafish *Pax6.2a*. A) rtPCR for *Pax6.2a* using cDNA from one to five dpf zebrafish embryos showing expression of the gene at all stages. rtPCR using *Islet-1*cDNA was used as control. B) Protein alignment of Pax6.2 from multiple species showing homology between the Pax6.2 proteins. Homology is highest in the homeobox but extends to several motifs in the PST domain. Zebrafish Pax6.2a shares highest homology with Pax6.2b proteins from other teleosts. (C–I) RNA *in situ* hybridisation analysis on fixed zebrafish embryos from 1 dpf to 5 dpf. C) At 24 hpf staining is seen in the head region of embryos. D) By 48 hpf expression has become limited to the developing retinae. E,F) Optical Projection Tomography (OPT) stills reveal the restricted expression of *Pax6.2* in the retina where it appears limited to the inner nuclear layer. G–I) Expression in the inner nuclear layer of the retina is maintained at 3 dpf (G), 4 dpf (H) and 5 dpf (I). J) Morpholino depletion of *Pax6.2a* gives rise to zebrafish embryos with relatively smaller eyes compared to embryos injected with a control morpholino. Embryos were fixed at 2 dpf and tested for the presence of *Pax6.2* transcript by *in situ* hybridization. K) Quantification of eye size reveals a 30% reduction in eye size relative to total body length in *Pax6.2a* morphants versus control morpholino injected embryos.

The regulated expression of *Pax6* in mammals depends on a wide genomic domain containing a large number of *cis*-regulatory elements [22]–[28]. Several of these are located at large distances from the promoters and coding region of the gene [24], [25], [26]. The distant downstream region in particular has been shown to be essential for *Pax6* expression as its heterozygous removal through deletion or translocation leads to the eye malformation aniridia in human patients [30], [31], [25], [64]. Alignments of the wider *Pax6* locus between mammalian and fish *Pax6.1* loci show conservation of most proximal CNEs, but absence of many of the distal ones in fish. We considered whether the absence of clear sequence conservation in teleost fish at the relative position of a conserved element in tetrapods would indicate the loss of that element in fish, or the gain of that element in the tetrapod lineage. To help distinguish between these possibilities we set out to obtain an outgroup for comparative studies. The elephant shark is a cartilaginous fish with a relatively small genome, situated at the base of the jawed vertebrate lineage, having split off from the bony vertebrate lineage before the split between the ray-finned fish and tetrapods [65]. We searched the 1.4× coverage sequence of the elephant shark genome [66] for the *Pax6* gene by BLAST. To our surprise the search identified two scaffolds each containing a different *Pax6* fragment. PCR primers were designed for these fragments and used to identify positive BAC clones (23H6 and 37E6) from a genomic BAC library. After complete sequencing the BAC clones were found to encode two different *Pax6* genes belonging to separate gene loci (Figure 3A). The first elephant shark *Pax6* (hereafter referred to as *Pax6.1*) gene is highly homologous to the mammalian *Pax6* gene, both in terms of gene structure and protein conservation (Figure 3B, 3C). The second elephant shark *Pax6* gene lacks the N-terminal exons of the canonical *Pax6* gene and encodes a Pax6 protein lacking the paired-box DNA-binding domain (Figure 3B, Figure S2). We have termed this novel paired-less *Pax6* gene as *Pax6.2*. We next used the genomic sequence to design probes to rescreen our BAC library and walk outward from the *Pax6*-containing BACs. In total we obtained 477 kb of genomic sequence for the *Pax6.1* locus and 156 kb of sequence for the *Pax6.2* locus. A number of flanking genes were present in the BAC contigs (Figure 3A) and allowed analysis of synteny around the genes. Synteny around the elephant shark *Pax6.1* gene is fully conserved to the human *PAX6* locus, with all genes present in our BAC contig found in equivalent positions to their order in the mammalian locus (Figure 3A, Figure 7B), clearly indicating a common ancestry of elephant shark *Pax6.1* and mammalian *Pax6*. The elephant shark *Pax6.2* gene is adjacent to a reticulocalbin gene (*Rcn3*), but none of the other genes in the *Pax6.2* locus is syntenic with the mammalian *Pax6* locus (Figure 3A, Figure 7D). Next we looked for tissue-specific expression of elephant shark *Pax6.1* and *Pax6.2*. rtPCR analysis using RNA from 13 different adult elephant shark tissues revealed expression of *Pax6.1* in brain, eye and pancreas, with a weaker signal in intestine, in accordance with the expression of *Pax6.1* in other organisms. Elephant shark *Pax6.2* has a more limited expression in the eye and kidney (Figure 3D). In mammals a paired-less Pax6 protein isoform, equivalent to the elephant shark *Pax6.2*-encoded protein, is produced from an internal promoter (Pα)in intron 4 of the single *Pax6* gene [38]. To determine whether the existence of the *Pax6.2* gene in elephant shark would be to act as substitute for the internal transcript of the mammalian gene (or vice versa) we investigated whether a similar α-transcript is also produced from the elephant shark *Pax6.1* gene. We found an elephant shark *Pax6.1*α transcript in the brain and eye, but not in pancreas (Figure 3E). While the expression of *Pax6.2* in the eye is unsurprising for a *Pax6* homolog, it is intriguing to see expression in the kidney, suggesting a novel function has been acquired by elephant shark *Pax6.2* in this tissue.

### An ancient conserved element identifies a novel long-range enhancer

Multispecies sequence alignments with the elephant shark *Pax6.1* genomic sequence reveal a large number of highly conserved non-coding elements in the locus. In accordance with a general observation [57] the number and conservation level of non-coding elements around *Pax6* is significantly higher when comparing the elephant shark *Pax6.1* and mammalian *Pax6* locus, than when either is compared to the teleost *Pax6.1* loci. In particular, clear conservation with elephant shark is observed for several mammalian long-range CNEs that appear absent in teleosts, both in the distal upstream and downstream regions, indicating these are ancient non-coding elements that have been secondarily lost in teleosts (Figure 3F).

**Figure 4:**
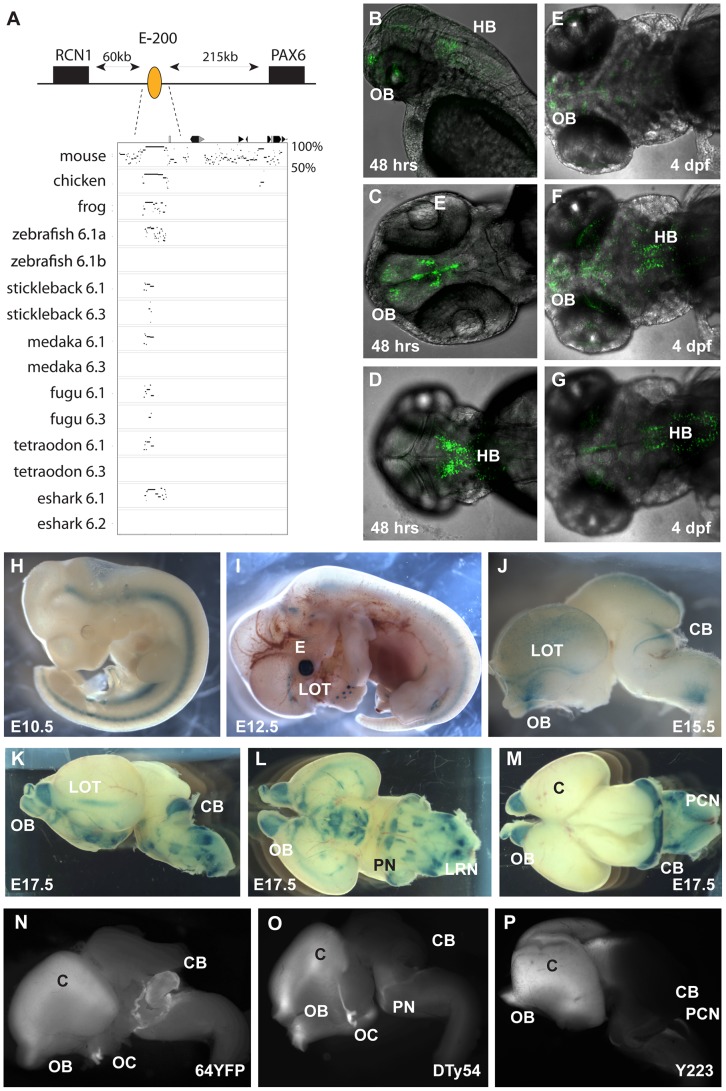
Characterization of the E-200 CNE in mouse and zebrafish reporter transgenics. A) The E-200 CNE is a deeply conserved *cis*-element found upstream of all vertebrate *Pax6.1* loci and located about 200 kb upstream of human *Pax6*. A shorter stretch of the element is also conserved in the *Pax6.3* loci of fugu and stickleback. (B–G) Fluorescent reporter transgenic zebrafish with the human E-200 element. B) Lateral view of an E-200-gata2-GFP reporter line showing expression in the olfactory bulbs (OB) and hindbrain (HB). C) Confocal image of the ventral head region of a 48 hpf transgenic fish reveals expression in the olfactory bulbs and the proliferative zones of the ventral brain region. D) Confocal view of the dorsal embryo head showing expression in the hindbrain. (E–G) Ventral, medial and dorsal confocal views show GFP signal in the olfactory bulbs, lateral olfactory tracts, forebrain (E, F) and hindbrain (F, G) of a 4 dpf reporter transgenic fish. (H–P) LacZ reporter transgenic mice with the human E-200 element. (H) At E10.5 no enhancer specific expression is observed. Signal in the neural tube is intrinsic to the hsp68-LacZ cassette, and in the genital ridge is aspecific. I) From E12.5 expression starts to appear in the olfactory tracts at the base of the cortex. J) At E15.5 staining is seen in the olfactory bulbs (OB), the lateral olfactory tracts (LOT) and a thin band in the dorsal cerebellum (CB). Expression is also seen in the precerebellar neuro-epithelium (PCN), migratory streams and the lateral reticular nuclei (LRN) of the hindbrain. K) This pattern is maintained at E17.5, with increased cerebellar expression covering the full width of the organ. L) Ventral view of an E17.5 dissected brain shows expression in the lateral olfactory tracts and in the precerebellar nuclei including the pontine gray nuclei (PN). Staining in hypothalamic areas was not seen in other transgenic lines, while expression in the olfactory bulbs varied in strength between lines. M) Dorsal view of an E17.5 brain shows the expression in the cerebellum and precerebellar neuroepithelium. N–P) Comparison of reporter YAC transgenic mice carrying 420 kb of the human *PAX6* locus, which does not extend to include the E-200 long-range enhancer, to a targeted reporter insertion into the endogenous *Pax6* locus shows a deficiency of reporter expression in the cerebellum of the YAC reporter lines. N) Fluorescence of a YFP reporter integrated into the endogenous mouse *Pax6* gene is found in the olfactory bulbs (OB), cortex (C), optic chiasm (OC), cerebellum (CB) and pontine migratory stream (PMS). While reporter fluorescence signal of comparable strength is seen in both (O) single copy or multi-copy (N) YAC transgenic lines carrying 420 kb of the human *PAX6* locus in the olfactory bulbs, cortical lobes, optic chiasma and pontine migratory streams, expression is absent from the cerebella of these transgenics.

We focussed on the region upstream of *Pax6*. As all extragenic aniridia patient breakpoints identified so far disrupt the *Pax6* downstream region [32], [67], regulatory activity in the upstream region has remained poorly investigated. Comparative sequence analysis between mammals and the elephant shark *Pax6.1* locus reveals the presence of a number of long-range CNEs in this region. Many of these CNEs are not found in teleosts, and are therefore candidate *Pax6* enhancers that have been lost in fish. Analysis of these CNEs will be described elsewhere. Here we characterize one element of particularly prominent sequence conservation, located approximately 200 kb upstream of human *Pax6*, which has been named E-200. The E-200 element displays strong sequence conservation between elephant shark and human, but is also detected in the *Pax6.1* loci of several teleosts. Moreover, in fugu and stickleback a low level of sequence conservation is also seen in the *Pax6.3* loci (Figure 4A, Figure S3). The location of the element upstream of *Pax6*, between *Pax6* and *Rcn1*, suggests *Pax6* as its most likely target gene, but synteny conservation around the *Pax6.1* locus leaves the possibility that other genes in the region, notably *Wt1* and *Rcn1*, might be the actual targets. The conservation to several fish duplicate loci provides an opportunity to check linkage between the element and these genes. We found that in all gene loci containing the E-200 element, it was linked to *Pax6*, while linkage to *Wt1* or *Rcn1* was only found where *Pax6* and *Wt1* or *Rcn1* are both present (not shown). To determine the function of this element we first made transgenic zebrafish, using the human element. We observed expression of the fluorescent reporter in the olfactory bulbs, olfactory tracts and the hindbrain regions of transgenic embryos (Figure 4B–4G). Next we generated transgenic mice with the human E-200 element. At early developmental stages (E9.5–E11.5) no consistent staining pattern was seen. From E12.5 expression appeared in the olfactory tract regions and the upper rhombic lip of transgenic mice (4 expressing/10 total transgenic). The staining in the eye in Figure 4I was not consistent between all expressing lines and is most likely due to site of integration. By E15.5 expression was found around the olfactory bulbs, in the lateral olfactory tracts (LOTs) and started to appear in the cerebellum (Figure 4J). At E17.5 strong staining was seen in the cerebellum, as well as around the olfactory bulbs and LOTs. Staining was also observed in the precerebellar neuroepithelium (PCN), migratory streams and precerebellar nuclei (Figure 4K–4M). The E-200 element is a long-range enhancer located around 200 kb upstream of the *PAX6* P0 promoter. It is not present on the human YAC that was used in previous reporter transgenic studies on the long range regulation of *PAX6* [24]. Reporter fluorescence in the cerebellum of all transgenic lines carrying the YAC (Figure 4O, 4P) is weak or absent compared to the expected level as demonstrated by the fluorescence from a targeted insertion of YFP into the endogenous *Pax6* gene (Figure 4N; Kleinjan et al. in preparation). This suggests the presence of the long-range E-200 element is required to achieve appropriate expression of *Pax6* in the cerebellum. In contrast, fluorescence in other sites of E-200 activity is not noticeably lower in the YAC transgenics, suggesting sufficient redundancy of *cis*-regulatory activity in these tissues (Figure 4N–4P). The importance of the E-200 enhancer for cerebellar expression is in accordance with the conservation of this ancient enhancer linked to *Pax6* since early vertebrate evolution.

### The second *Pax6* locus in the elephant shark genome

**Figure 5:**
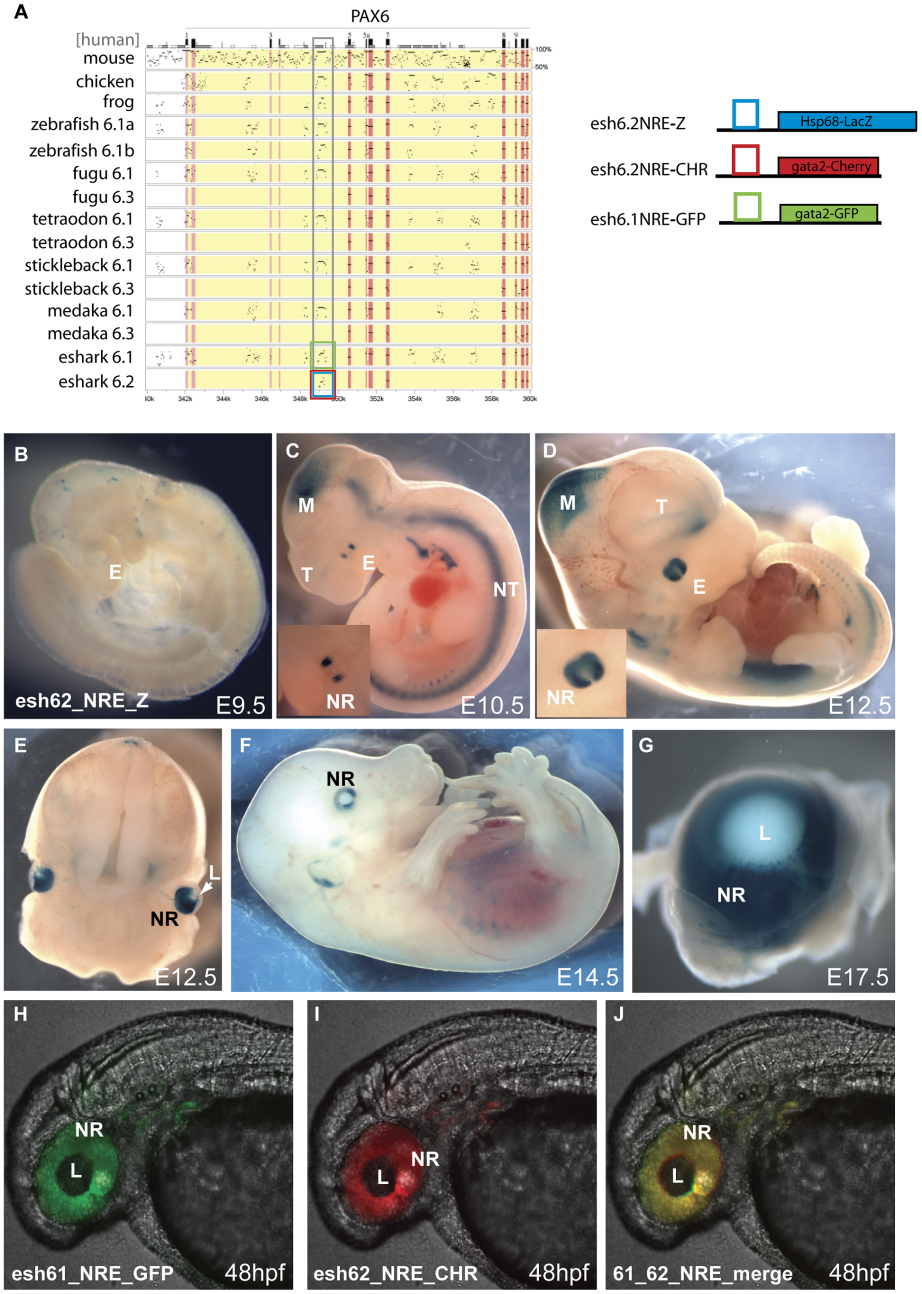
Transgenic analysis of the elephant shark Pα promoter/neuroretinal enhancer (NRE) element. A) PIP plot of the *Pax6* intragenic region shows the conservation at the Pα/NRE in intron 4 of mammalian *Pax6* in both *Pax6* loci in elephant shark. The NRE(Cm6.2) element is the only conserved non-exonic sequence between the *Pax6.1* and *Pax6.2* loci. To allow comparison with the mouse neuroretinal enhancer (NRE) LacZ reporter transgenic mice were made with the Cm6.2 element. (B–G) LacZ staining is seen in the neuroretina of these mice from E10.5 in a pattern that is highly similar to expression driven by the mouse element. B) No expression is seen at E9.5. C) Expression starts at E10.5 at the lateral sides of the developing eyes (E). Expression in the midbrain (M) is due to site of integration and is not seen in other lines. D) By E12.5 expression has widened to include the whole retina. The inset shows a larger view of the eye. E) Cross section through a E12.5 embryo head shows staining in the neuroretina (NR) and optic nerve. F) Expression continues at E14.5 in the neuroretina, and G) is maintained at E17.5 in the retina but not in the lens. (H–J) To test for functional conservation of the elephant shark *Pax6.1* and *Pax6.2* NRE we assessed the elements in zebrafish transgenics using the dual colour reporter system. H) The NRE element from the elephant shark *Pax6.1* locus drives GFP reporter expression in the retina of transgenic zebrafish at 48 hpf. I) The NRE element from the elephant shark *Pax6.2* locus similarly drives mCherry in the neuroretina at 48 hpf. J) The merged image shows functional equivalence of the Cm6.1 and Cm6.2 NRE elements in driving reporter expression in the zebrafish retina. NR, neuroretina, L, lens.

The elephant shark *Pax6.1* locus with its high level of sequence homology and synteny conservation to the mammalian *Pax6* loci has provided insight into the ancient history of the *Pax6 cis*-regulatory domain. In addition to the *Pax6.1* locus, our elephant shark BAC library screening yielded a BAC contig containing a second *Pax6* locus. The BACs were fully sequenced to reveal a novel *Pax6* homolog lacking the N-terminal exons that encode the paired box in canonical Pax6 isoforms. This second elephant shark *Pax6* gene was named *Pax6.2*. We aligned genomic sequence from the *Pax6.2* locus with other *Pax6* loci to identify putative CNEs in the locus. This revealed one clear CNE with conservation to the *Pax6.1* locus (Figure 5A). This element is located upstream of the *Pax6.2* gene but has homology to the intronic neural retina enhancer (NRE) located in intron 4 of canonical *Pax6.1* [27], [68]. Interestingly, in addition to retinal-specific enhancer activity the NRE element also serves as an internal promoter (Pα) in the mouse and zebrafish *Pax6.1* genes, and transcripts initiated from this promoter give rise to paired-less Pax6 isoforms in these species [38]; [37]. To test whether the elephant shark *Pax6.2* CNE (eshark6.2NRE) has enhancer activity we generated a number of mouse transgenic reporter lines with the element driving LacZ expression (4 expressing/8 total transgenics). Staining was found in the developing retina from E9.5 onwards in a pattern that closely resembles the expression of the murine NRE element [27]. No staining is seen at E9.5 (Figure 5B). Expression starts at E10.5 in two lateral domains on either side of the optic cup (Figure 5C), spreading wider from E11.5 (Figure 5D) to the full developing retina from E12.5 (Figure 5E), and continuing in the retina at E14.5 and E17.5 (Figure 5F, 5G). To assess the functional equivalence of the conserved NRE elements from the elephant shark *Pax6.1* and *Pax6.2* loci, we produced transgenic zebrafish with the CNEs driving GFP and mCherry fluorescent reporter genes. Both CNEs drove a clean and overlapping expression pattern in the retina (Figure 5H–5J). This confirms the elephant shark *Pax6.1* and *Pax6.2* NREs as neuroretinal enhancers that derive from an ancestral element that was present before the duplication event that created the elephant shark *Pax6.1* and *Pax6.2* loci.

### Phylogenetic and synteny relationships between vertebrate *Pax6* loci

**Figure 6:**
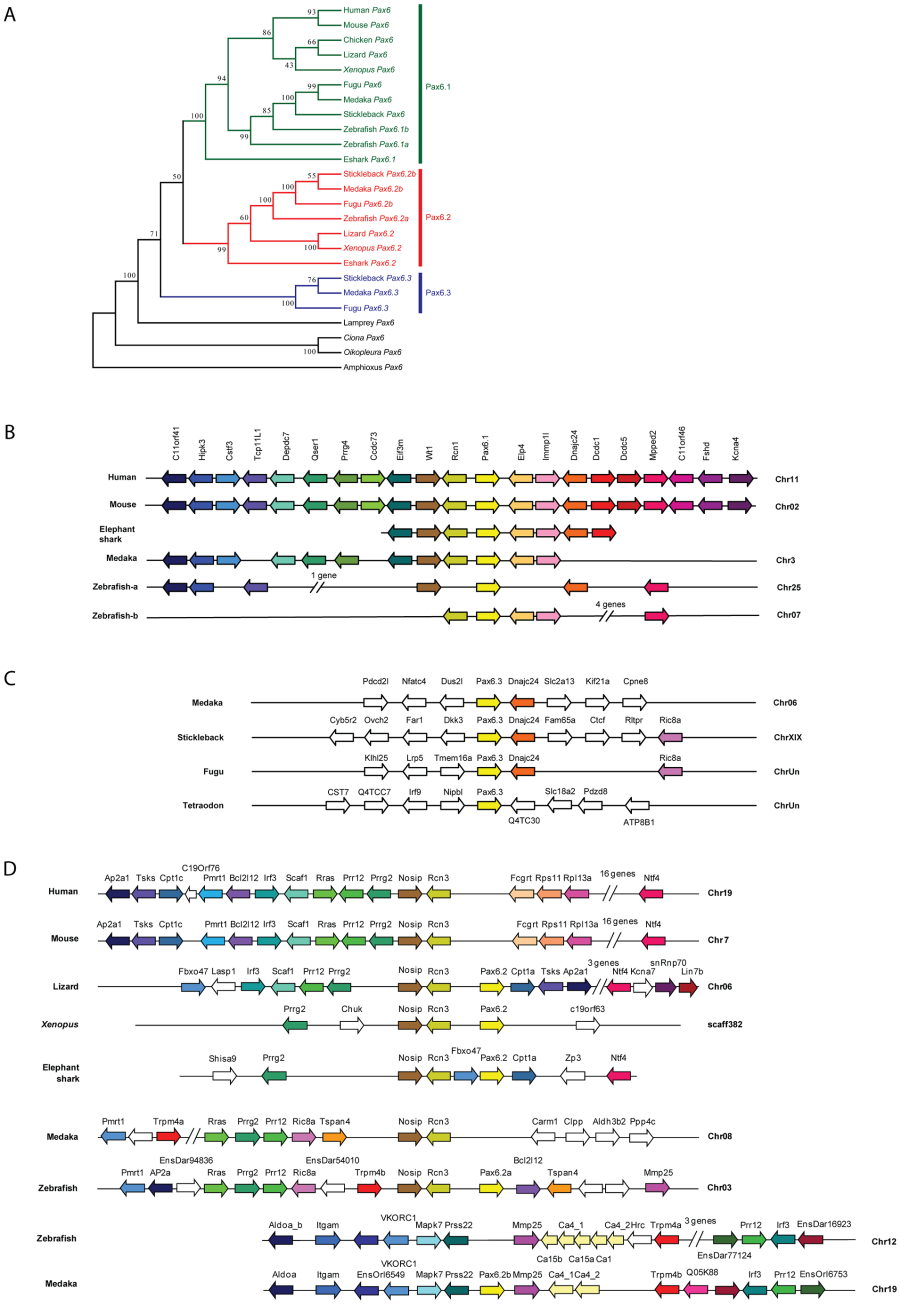
Phylogenetic and syntenic relationship between vertebrate *Pax6* loci. Phylogeny of the *Pax6* gene family is corroborated by comparison of gene content in the synteny regions and provides insight into the ontogeny of the *Pax6* loci. A) A Neighbor-Joining (NJ) tree of *Pax6* genes from several vertebrates and few invertebrates shows a clear grouping of jawed vertebrate *Pax6* genes into three clades: *Pax6.1*, *Pax6.2* and *Pax6.3*. The tree was generated using MEGA5 with a Poisson substitution model. Values at the nodes represent bootstrap percentages obtained from 1000 replicates. B) The *Pax6.1* gene resides in an ancient synteny block that is perfectly conserved from elephant shark to human. Gene content is also largely conserved in the *Pax6.1* upstream region in teleost fish, while synteny breaks beyond the *Immp1l* gene. The duplicate zebrafish *Pax6.1* loci show a clear sub-partitioning of the genes in the syntenic region. C) *Pax6.3* is only found in acanthopterygian fish and forms a mini block of conserved synteny with the *Dnajc24* gene. D) The paired-less *Pax6.2* gene is found in a region of conserved synteny with the *Rcn3* and *Nosip* genes in the elephant shark, *Xenopus* and lizard genomes. In mammals there is no *Pax6* adjacent to *Rcn3* and *Nosip*. Reciprocal *Pax6.2* duplicates have been retained in teleosts. Zebrafish *Pax6.2a* is found in synteny with *rcn3* and *nosip*, but the *rcn3*/*nosip* synteny region in medaka does not contain *Pax6*. Instead a *Pax6.2* (*Pax6.2b*) is found between *prss22* and *mmp25* in acanthopterygians, where conversely it is absent from this region in zebrafish. *Rcn1*, reticulocalbin 1, *Elp4*, elongator protein subunit 4, *Wt1*, Wilms tumour 1, *Immp1l*, inner mitochondrial membrane peptidase like 1, *Dnajc24*, dna J homolog, subfamily C, member 24, *Mmped2*, metallophosphoesterase domain containing 2, *Rcn3*, reticulocalbin 3, *Nosip*, nitric oxide synthase interacting protein, *Fcgrt*, Fc fragment of IgG, receptor, transporter, alpha, *Prss22*, protease serine 22, *Prr12*, proline rich 12, *Irf3*, interferon regulatory factor 3; *Mmp25*, matrix metallo peptidase 25, *Trpm4*, transient receptor potential cation channel, subfamily M member 4, *Ca4*, carbonic anhydrase IV; *Mapk7*, mitogen activated protein kinase 7, *Ric8a*, resistance to inhibitors of cholinesterase 8 homolog A.

The existence of a second, paired-less *Pax6* gene in elephant shark suggested two possibilities: a cartilaginous fish-specific duplication event after the split from the bony vertebrate lineage; or an ancient duplication event that occurred in a gnathostome ancestor. We reasoned that if the duplication had occurred in a gnathostome ancestor, we might be able to find orthologs of this paired-less *Pax6* gene in some species of bony vertebrates. We therefore searched the genomes of bony vertebrates for additional *Pax6* loci with similarities to elephant shark *Pax6.2* in gene structure, locus synteny and *cis*-element content. Remarkably we found novel paired-less *Pax6* genes in the genomes of frog (*Xenopus tropicalis*), Anolis lizard and teleost fishes (zebrafish, stickleback, medaka, and fugu). Evidence for the existence of *Pax6.2* orthologs was also found in cod, sea bass and Nile tilapia (data not shown). Despite extensive searches, no *Pax6.2* gene was found in birds or mammals. Thus in many fish species three *Pax6* genes exist, the canonical *Pax6.1* as well as the newly identified *Pax6.2* and re-defined *Pax6.3* genes. The zebrafish genome also harbors three *Pax6* genes, but in this case the trio consists of the fish specific duplicates of canonical *Pax6.1* (*Pax6.1a* and *Pax6.1b* [58], [59]) and the novel paired-less *Pax6.2* gene. To visualize the relation between the members of the newly defined *Pax6* gene family we generated a phylogenetic tree using the Neighbor Joining method [69]. This phylogenetic tree supports the classification of gnathostome *Pax6* genes into three clades, with zebrafish *Pax6a* and *Pax6b* both in the *Pax6.1* clade. The paired-less *Pax6.2* genes form their own clade and the acanthopterygian *Pax6.3* genes make up the most distant clade (Figure 6A). Comparison of synteny relationships around the *Pax6* family members of different species provides further insight into the divergence of the gene loci. The well known synteny block around the mammalian *Pax6* gene is fully conserved in the elephant shark *Pax6.1* contig. Synteny is also largely conserved in the *Pax6.1* loci of acanthopterygian fishes, but a subpartitioning of genes is observed at the zebrafish *Pax6.1a* and *Pax6.1b* loci (Figure 6B). In contrast, synteny conservation around the *Pax6.3* gene in acanthopterygians is limited and only encompasses the adjacent *Dnajc24* gene in medaka, fugu and stickleback (Figure 6C). Closer examination of the *Dnajc24* genes reveals that conservation between orthologs from the same locus is higher than across loci. In particular, zebrafish *Dnajc24* is more related to mammalian *Dnajc24* than to medaka or stickleback *Dnajc24*. Synteny around the elephant shark *Pax6.2* locus and the *Xenopus*, lizard and zebrafish loci provides evidence that they are of common descent. In these species the *Pax6.2* gene is found in close synteny with *Rcn3* (reticulocalbin 3), *Nosip* (nitric oxide synthase interacting protein), *Prrg2* (proline rich G-carboxyglutamic acid 2) and *Cpt1* (Figure 6D). No *Pax6.2* ortholog was found in the *Nosip*/*Rcn3* locus in birds or mammals (Figure 6D). In acanthopterygian fish species the *Pax6.2* gene is also absent from the *Nosip*/*Rcn3* locus, but in these species a *Pax6.2* ortholog is present adjacent to the *Mapk7*, *Prss22* and *Mmp25* genes. The locus also harbors *Trpm4*, *Irf3* and *Prr12* paralogs suggesting a link with the *Nosip/Rcn3* locus. This locus is well conserved in zebrafish, except for a lack of *Pax6.2*. These observations strongly suggest that after the FSGD duplicate copies of the *Pax6.2* gene (*Pax6.2a* and *Pax6.2b*) must have persisted for some time in the basal teleost genome until after the split between the zebrafish and acanthopterygians lineages. Subsequently, one copy of the gene was lost reciprocally from the two loci in zebrafish and the acanthopterygians. In zebrafish, *Pax6.2a* was retained alongside *Rcn3* and *Nosip*, while in acanthopterygians the reciprocal duplicate (*Pax6.2b*) was retained in the more derived locus between the *Prss22* and *Mmp25* genes (Figure 6D, Figure S4).

### Characterisation of the novel zebrafish *Pax6.2* gene

Having identified a *Pax6.2* homolog in zebrafish we performed rtPCR for this novel *Pax6* gene to check for expression. cDNA was generated from zebrafish embryos covering the first five days post fertilization (1–5 dpf). rtPCR results show that *Pax6.2* is expressed in zebrafish embryos at all stages examined. rtPCR for *Islet-1*, known to be expressed at these stages was used as control (Figure 7A). Alignment of Pax6.2 proteins from various species shows clear homology between the genes, but indicates a higher similarity between zebrafish *Pax6.2* and the acanthopterygian *Pax6.2* genes (Figure 7B). To assess the tissue-specific expression pattern of zebrafish *Pax6.2* we performed RNA *in situ* hybridization analysis on fixed zebrafish embryos from 1 dpf to 5 dpf. The staining pattern reveals *Pax6.2* to have a highly restricted expression. At 24 hpf staining is seen more widely in the head region of embryos (Figure 7C). The expression becomes limited to the developing retinae only from the next stages examined (48 hpf to 5 dpf) (Figure 7D, 7G–7I). We used Optical Projection Tomography (OPT, [70]) to visualize the *in situ* expression pattern at 2 dpf, which confirmed the restricted expression of *Pax6.2* in the retina only (Figure 7E, 7F, Video S1), where it appears limited to the inner nuclear layer, potentially marking the amacrine cells. Finally we performed morpholino knock-down experiments to investigate the potential function of *Pax6.2*. Injections of a *Pax6.2* morpholino into zebrafish oocytes resulted in zebrafish embryos with relatively smaller eyes compared to embryos injected with a control morpholino (Figure 7J). To quantify this observation we titrated the morpholino concentration and repeated the injections with the optimal dose of *Pax6.2* and control morpholinos. Embryos were fixed at 2 dpf and tested for the presence of *Pax6.2* transcript by *in situ* hybridization. *Pax6.2* ISH signal was unaffected in control morpholino injected embryos. The majority of *Pax6.2* morpholino injected embryos had completely lost *Pax6.2* according to ISH signal, while the remainder showed partial ISH signal loss. We measured the eye diameter of *Pax6.2* morphants relative to total body length of the embryos. A clear deficiency in eye size was seen in the *Pax6.2* morphants in comparison with control morpholino injected embryos, indicating an essential role for the paired-less Pax6.2 protein in eye development (Figure 7K, Figure S5).

**Figure 8:**
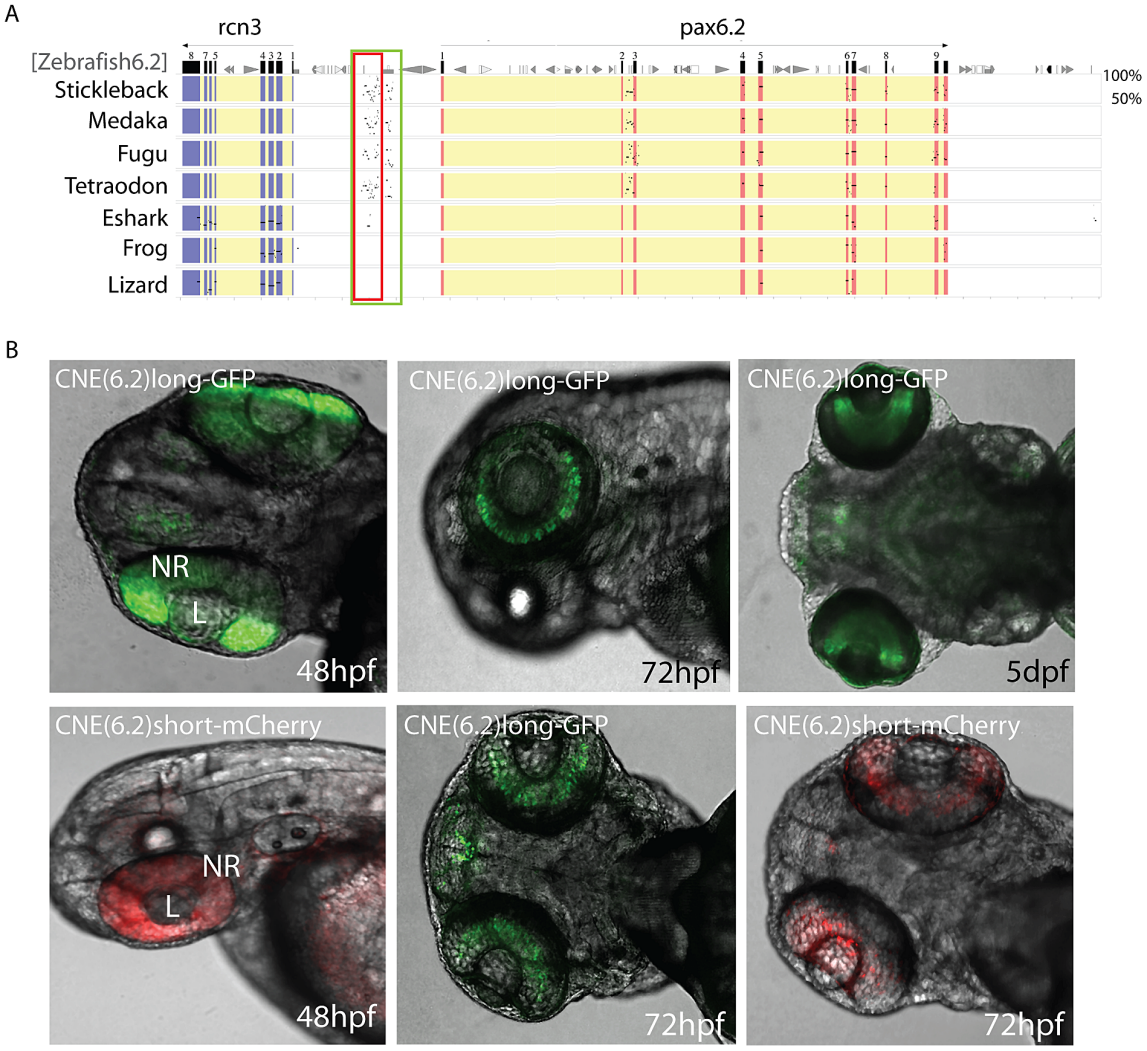
Identification of a novel enhancer specific for the inner nuclear layer of zebrafish retina. A) Sequence alignment of the *Pax6.2* genomic region with the *Pax6.2* loci from multiple species reveals a region of clear sequence conservation upstream of the gene. A wide fragment of homology is found between zebrafish *Pax6.2* and the acanthopterygian fish, with a smaller stretch of conservation to the elephant shark *Pax6.2* locus. No conservation is seen with the frog and lizard loci. B) The zebrafish *Pax6.2* CNE was cloned as a longer fragment covering the teleost conservation and as a shorter fragment centered on the elephant shark conserved sequence. Dual colour fluorescence transgenesis with both fragments produced a highly specific expression pattern in the retina at 48 hpf, and became restricted to the inner nuclear layer of the retina at 72 hpf and 5 dpf. NR, neuroretina, L, lens.

Finally, we performed sequence alignments using PipMaker to identify CNEs in the *Pax6.2* gene loci. A distinct region of sequence homology outside the exons of the gene was found in the upstream regions of zebrafish, stickleback, medaka and fugu (Figure 8A). Conservation of the CNE between zebrafish and the other teleosts is seen despite their origin from reciprocal *Pax6.2* duplicate loci. A smaller sub-region of the CNE also showed conservation to the elephant shark *Pax6.2* upstream region. Intriguingly, this short conserved fragment maps to the edge of the elephant shark *Pax6.2* NRE element, but has no distinguishable homology to the NRE sequence in intron 4 of *Pax6.1* genes. To examine the putative functional activity of the *Pax6.2* CNE region, a longer fragment covering the homology region of the teleost CNEs, and a shorter fragment centered around the zebrafish to elephant shark homology region, were cloned from the zebrafish *Pax6.2* locus and inserted into fluorescence reporter constructs for the production of transgenic fish. Fluorescence was seen in the retinae of transgenic fish at 72 hpf with both the long (GFP) and short (mCherry) fragments (Figure 8B). In addition the long fragment showed some minor expression in the forebrain region in a subset of transgenics. Fluorescence signal was restricted to the inner nuclear layer (INL), in accordance with the RNA *in situ* pattern of *Pax6.2* (Figure 7G). This suggests that sequence divergence at the NRE in separate branches of the *Pax6* gene family has led to subtle differences in the spatial detail of its retinal enhancer activity. It confirms the ancient role of the NRE element as a retinal enhancer in the ancestral *Pax6* locus. In summary, we have discovered a novel, paired-less *Pax6* gene in the genomes of multiple species. In zebrafish *Pax6.2a* is expressed in the inner nuclear layer of the retina and we have identified a conserved enhancer driving this expression pattern.

## Discussion

The genome is a remarkable repository of biological information. Within its sequence it contains not only a complete set of instructions for embryonic development of the organism, maintenance of adult homeostasis and the response to environmental interactions, but also a record of the evolutionary history of its genes and associated sequences. By comparison of genomic sequences from multiple contemporary species of divergent lineages attempts can be made to reconstruct the ontogeny/phylogeny of specific genes and their regulatory landscapes. It is well established that early vertebrate evolution was accompanied by two rounds of whole genome duplications (2R) [44], while a third round (3R) has occurred later specifically in the teleost lineage of bony fish around 320 million years ago [40]. Duplication events are recognized as powerful drivers of evolutionary change as they provide enhanced opportunity for the subsequent modification or mutation of gene duplicates, and/or the alteration of their *cis*-regulatory landscapes, while the other copy maintains critical functions under selective pressure [45], [48].

We have previously shown that divergence of the *cis*-regulatory landscapes around the duplicate genes of the developmental regulator *Pax6* in the zebrafish has led to their subfunctionalization [59]. In this study we demonstrate that duplicate *Pax6* loci also exist in other vertebrate species. Unexpectedly, we have uncovered that these duplicates form a diverse family of *Pax6* genes that are derived from multiple independent duplication events. These duplications were followed by multiple independent gene losses in separate vertebrate lineages, such that a variable subset of family members has been retained in contemporary vertebrate species. We show that while the genomes of mammals and birds contain only a single *Pax6* gene, other species have two (elephant shark, *Xenopus tropicalis*, Anolis lizard) or even three *Pax6* genes (zebrafish, stickleback, medaka, other teleost species). The various *Pax6* family members are characterized by differences in their protein structure and in the composition of their *cis*-regulatory landscapes.

In mammals a multitude of key functions in development and maintenance of the eye, brain and pancreas are carried out by a single *Pax6* gene [10]. A number of variant protein isoforms encoded by this single mammalian gene are thought to implement different, complementary subsets of *Pax6* activity [71]–[73]. The canonical Pax6 protein contains two DNA binding domains: an N-terminal paired domain followed by a paired-type homeodomain. A proline-serine-threonine rich transactivation domain is located at the C-terminus [13], [19]. An alternative isoform, Pax6(+5a) is made by inclusion of an alternatively spliced exon 5a resulting in a 14 amino acid insertion in the paired domain leading to recognition of a different binding sequence [35]. A third isoform, Pax6ΔPD, produced from a transcript initiated at an internal promoter (Pα) located in intron 4 of the gene lacks the entire paired domain [27], [38]. Reporter transgenic studies of a BAC engineered to express dsRed from the Pα promoter have shown that Pax6ΔPD is expressed in a highly restricted expression pattern [36], [37]. In mouse Pax6ΔPD is found in the retina and olfactory bulbs, while in the zebrafish it is only seen in the amacrine cells in the retina. The role of this isoform is currently unclear.

Inspection of the *Pax6* genes of several ray-finned fish species indicated that the ability to encode the alternative exon 5a is present only in the *Pax6.1* genes of the fish species examined. Sequence comparison between the 5a exons from multiple species shows that it is less conserved than the rest of the paired-box, both in amino acid composition and length (Figure 1A), suggesting that the main function of this peptide might be disruption of the paired box, putting less stringency on the actual sequence itself. In contrast, the other *Pax6* copies of the teleost species examined completely lack the alternative exon 5a. The exception is zebrafish where both *Pax6* duplicates have a well conserved exon 5a. Moreover, the conspicuous sub-partitioning of CNEs between the duplicate zebrafish *Pax6a* and *Pax6b* loci is not seen in the multiple gene loci of medaka, stickleback, *Tetraodon* and pufferfish, where instead the loci are devoid of any recognizable sequence conservation between them outside of the exons of the *Pax6b* loci, apart from a short conserved fragment at the E-200 *cis*-element. Thirdly, in zebrafish the ubiquitous *Elp4* gene has been retained next to one of the *Pax6* copies (*Pax6.1b*) while the long-range enhancers of the DRR have mostly been conserved in the other copy (*Pax6.1a*) [59]. In the acanthopterygians both the *Elp4* gene and the long-range enhancers are found adjacent to the *Pax6.1* gene, while the other *Pax6* duplicate is located in a different synteny region. Taken together these observations strongly suggest that the *Pax6* duplicates of zebrafish and those of the other teleosts derive from different duplication events. The near total lack of CNE conservation suggests that the acanthopterygian *Pax6* duplicates have a more ancient evolutionary origin [57], [74]. Based on these observations we propose to refer to the zebrafish duplicates as *Pax6.1a*/*b* and refer to the acanthopterygian duplicates as *Pax6.1* and *Pax6.3*.

To gain more insight into the evolutionary origin of the fish *Pax6* loci we screened a BAC library from the elephant shark as an outgroup for comparative studies. Contrary to expectation we identified two separate *Pax6* loci in this cartilaginous fish, which we designated as *Pax6.1* and *Pax6.2*. The elephant shark *Pax6.1* locus is highly similar to the tetrapod *Pax6* locus and the *Pax6.1* loci of ray-finned fish. In contrast, the second elephant shark *Pax6* locus encodes a *Pax6* gene lacking the N-terminal exons of the canonical *Pax6* and is predicted to produce a *Pax6* homolog without the paired domain. It is thus similar to the Pax6ΔPD isoform derived from the internal Pα promoter of mammalian *Pax6*. The presence of a separate paired-less *Pax6* gene in the elephant shark genome suggested that it might be fulfilling the equivalent role of the Pα-derived mammalian paired-less Pax6 isoform. However, we show that a paired-less isoform is also produced from the elephant shark *Pax6.1* gene. Nevertheless, persistence of the gene suggests it does serve a unique function and it is possible that its specific expression not only in the eye but also in the kidney accounts for its retention in the elephant shark genome.

The identification of two *Pax6* gene loci in the elephant shark raised two possibilities: Either the duplication of the *Pax6* locus occurred uniquely in the cartilaginous fish lineage after the split from the bony vertebrate lineage, or the duplicate loci had arisen before the split between cartilaginous and bony fish lineages. We resolved this question by looking for potential *Pax6.2* homologs in non-cartilaginous species, using the genes in synteny with the elephant shark *Pax6.2* gene in our searches. Sequence analysis of our elephant shark *Pax6.2* BAC contig revealed the presence of an adjacent reticulocalbin gene, *Rcn3*, as well as the genes *Nosip*, *Prrg2*, *Cpt1a*. A search for loci containing these genes in other vertebrate genomes led us to the identification of novel *Pax6.2* orthologs in several species, including frog (*Xenopus tropicalis*), lizard (Anolis lizard) and many fish species including medaka, stickleback and zebrafish. This clearly indicates that the duplication that gave rise to the *Pax6.2* gene must have occurred before the split between cartilaginous and bony fish. Although the presence of a *Pax6.2* in teleosts means that the gene was present during the FSGD, we could only find a single *Pax6.2* gene in the fish genomes examined, indicating the second *Pax6.2* duplicate has been lost. Under the DDC model [46], [48] non-functionalization of one copy of gene duplicates is often seen for genes with a single function, and accordingly we show by *in situ* hybridization that *Pax6.2* has a highly specific expression restricted only to the neuroretina of developing fish embryos. Nevertheless we do find evidence for the original presence of duplicated *Pax6.2* genes in early teleost fish by comparisons of synteny around the zebrafish *Pax6.2* locus with synteny around the *Pax6.2* loci of medaka, stickleback and fugu. While in zebrafish *Pax6.2* is found in a synteny block with the *Nosip* and *Rcn3* genes in common with the elephant shark, frog and lizard, the gene is located in a different synteny block in medaka, stickleback and fugu. This indicates that reciprocal duplicate copies of the gene were lost in zebrafish versus the acanthopterygian species. Despite being reciprocal duplicates (which we refer to as *Pax6.2a* for zebrafish and *Pax6.2b* for the acanthopterygians), both gene loci share a well conserved CNE in their upstream region that also shows some homology to the elephant shark *Pax6.2* upstream region. In reporter transgenic zebrafish both the full-length fish CNE and a shorter fragment around the elephant shark-zebrafish conserved region drive highly specific expression in the inner nuclear layer of the retina in accordance with the RNA *in situ* pattern for *Pax6.2*. The retention of a *Pax6.2* gene in multiple species suggests functional importance and our morpholino knock-down experiment in zebrafish embryos demonstrates a role for *Pax6.2* (*Pax6c*) in eye development. Nevertheless the *Pax6.2* gene has eventually been lost in the avian and mammalian lineages. It is currently unclear whether this suggests a change in the molecular networks for eye development between species, or redundancy in the availability of a paired-less form of the Pax6 transcription factor. Despite the absence of *Pax6.2* a paired-less Pax6 isoform is produced in birds and mammals, generated from a transcript initiating at an internal promoter Pα in intron 4 of the canonical *Pax6.1* gene [37], [38]. Expression of this paired-less Pax6.1 isoform (pax6ΔPD) has been shown to be specific to the eye in zebrafish and mouse [38]. However, these alternative ways of producing a paired-less isoform are not mutually exclusive as the Pax6.1 paired-less isoform is also produced in zebrafish [37] and elephant shark (this study) which have nonetheless retained the *Pax6.2* gene.

The genomic region around mammalian *Pax6* contains a large number of *cis*-regulatory elements [22]–[28]. Functional constraint on these elements imposes a strong demand on the conservation of their sequence, and consequently many *cis*-regulatory elements can be identified as CNEs. However, significant divergence of regulatory sequence following duplication can occur with or without concomitant changes in expression pattern. In extremis this can result in conservation of functional activity despite the disappearance of recognizable sequence conservation [75]. Our analyses of reporter expression driven by individual CNEs versus the full zebrafish intron 7 sequences show that the regulatory activity residing in the intron is contributed by the 7CE2 and 7CE3 elements. Loss of the conserved sequences of the 7CE2 element from zebrafish *Pax6.1b* intron 7 correlates with absence of expression in the diencephalon, suggesting that regulatory activity is not maintained in the absence of sequence conservation. Curiously no specific expression was observed at the stages examined for the 7CE1 element despite its conservation in both loci, suggesting the 7CE1 CNE must fulfill some other, unknown function. In contrast, analysis of reporter expression driven by intron 7 of medaka *Pax6.3* in comparison with the patterns directed by the introns 7 of both zebrafish *Pax6.1a* and *Pax6.1b* loci and medaka *Pax6.1*, suggest that this region does show conservation of functional activity despite lack of sequence conservation. However, our new observations on the more ancient ontogeny of the *Pax6.1* and *Pax6.3* loci suggest that most enhancer elements have formed independently in the *Pax6.1* and *Pax6.3* loci, and that the presence of enhancers located in similar positions (e.g. intron 7) in the acanthopterygian *Pax6.1* and *Pax6.3* loci is most likely coincidental. The independent acquisition of *cis*-regulatory elements in the *Pax6.1* and *Pax6.3* loci, which would be predicted if the duplication of the loci occurred before the large-scale appearance of *cis*-regulatory elements during the early stages of gnathostome evolution [53]??[74], is supported by sequence alignments using stickleback *Pax6.3* as baseline, which reveal a number of *Pax6.3* loci-specific CNEs in addition to the 7CE(6.3) element as further candidate *Pax6.3* enhancers that are not found in the *Pax6.1* loci (Figure S1).

Sequence alignments between the three *Pax6* sub-family members support the independent acquisition of most *cis*-regulatory elements after the WGD events. The large number of CNEs conserved between mammalian and elephant shark *Pax6.1* loci indicates that the majority of *Pax6.1* enhancers must have appeared in the timeframe between the WGD and divergence of cartilaginous and bony fish, with few additional *cis*-elements having appeared since. However, we also show that a limited number of CNEs are present in multiple branches of the *Pax6* gene family. Comparisons of the elephant shark *Pax6.2* locus with multiple *Pax6.1* loci revealed a single conspicuous CNE. Intriguingly, the homologous element maps to the neuro-retina enhancer (NRE) in *Pax6.1* intron 4 that coincides with the internal Pα promoter [27]. We show that the sequence conservation of this CNE extends to function as reporter expression driven by the elephant shark 6.2 NRE element in transgenic mice and zebrafish is very similar to the retinal expression driven by the mouse NRE element [27]. Using transgenic zebrafish we show that both elephant shark 6.1NRE and 6.2NRE elements drive an identical expression pattern in the neuroretina. This conservation of sequence and function of the NRE indicates the element was already present in the ancestral gene locus before the WGD events that led to separate *Pax6.1* and *Pax6.2* genes.

Another ancient enhancer that must have been present before the 1R/2R WGD events is located in the region upstream of *Pax6*, between the *Pax6* and *Rcn1* genes. This highly conserved element, E-200, is present in all *Pax6.1* loci including the tetrapod, elephant shark *Pax6.1* and teleost *Pax6.1* loci. A small central fragment of this element is also conserved in two of the teleost *Pax6.3* loci, medaka and *Tetraodon* (Figure S1), and was subsequently also found in both *Pax6.1* and *Pax6.3* loci of the Nile tilapia. It is the only non-coding sequence conserved between the acanthopterygian *Pax6.3* loci and the fish/mammalian *Pax6.1* loci. Both mouse and zebrafish reporter transgenesis revealed expression from the E-200 element in the hindbrain/cerebellar region, olfactory bulbs and lateral olfactory tracts. Reporter expression in the cerebellum was of particular interest as we had previously noted that a large YAC reporter transgene was deficient in driving expression in the cerebellum of transgenic mice. As the *PAX6* genomic region contained in the YAC does not extend to the E-200 element we propose that the lack of cerebellar expression from the YAC is due to the absence of this element, though further long-range elements could also be involved. The presence of the E-200 CNE near *Pax6.3* clearly links the element to *Pax6* since the *Pax6.3* loci do not contain *Rcn1* or *Wt1*.

In summary, we demonstrate that the locus for the developmental regulator *Pax6* has undergone multiple duplication events, followed by variable divergence or loss of the gene duplicates. Divergences between the loci have affected both the coding region as well as the *cis*-regulatory landscapes in lineage specific ways, resulting in a family of variant *Pax6* genes in vertebrate genomes. The existence of multiple variant *Pax6* genes and proteins also occurs in other parts of the animal kingdom. In *C. elegans* two different classes of mutation, *vab-3* and *mab-18*, are known to affect different isoforms from the same *Pax6* gene. One of these, the *mab-18* isoform, encodes a paired-less isoform that causes a male fertility phenotype due to a sensory defect in the male sensory organ [76]. The *Drosophila* genome contains four *Pax6* orthologs, occurring in two sets of adjacent gene pairs. The *eyeless* and *twin-of-eyeless* pair encode the canonical Pax6 homologs, whose absence causes the eyeless phenotype [7], [77]. A second pair of *Pax6*-like genes, the *eyegone* and *twin-of-eyegone* gene pair, have an incomplete paired box and are considered to be the functional equivalent in *Drosophila* of the mammalian Pax6(+5a) isoform [78].

**Figure 9:**
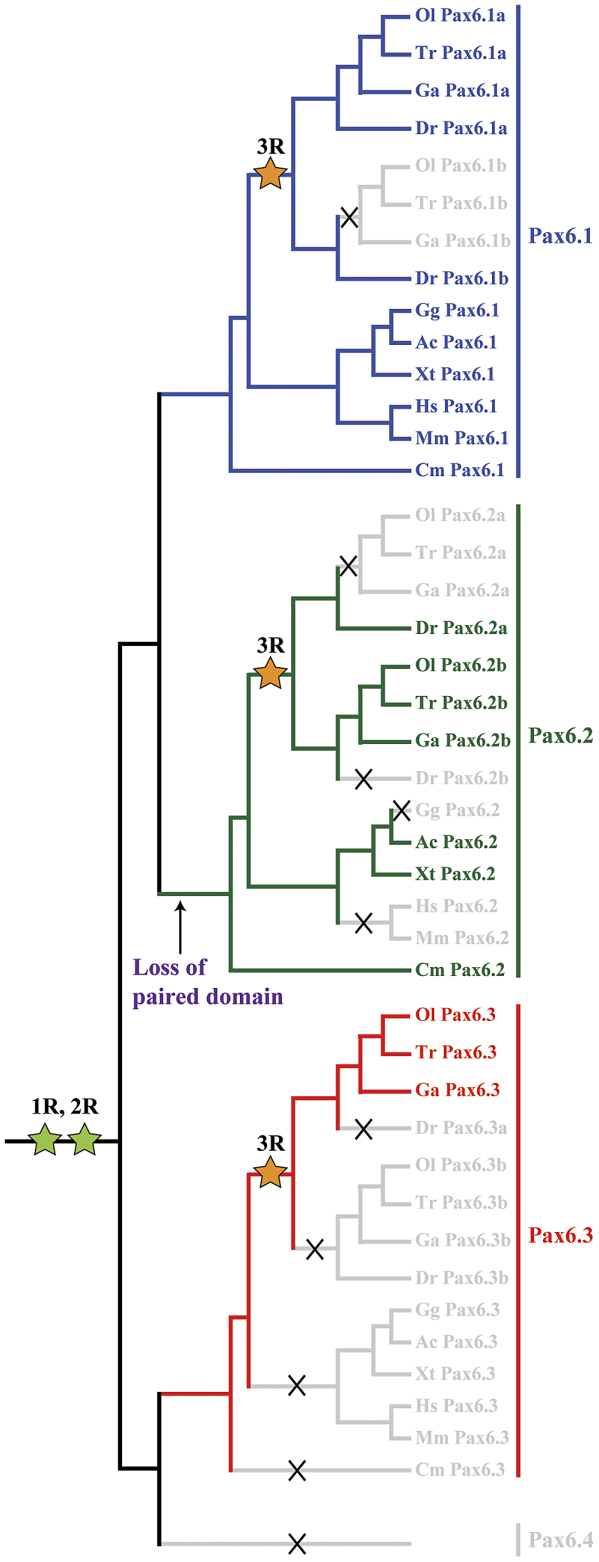
Evolutionary history of the *Pax6* gene family in vertebrates. Two rounds of whole genome duplication (WGD (1R, 2R)) at the base of vertebrate evolution gave rise to four *Pax6* genes (*Pax6.1*–*Pax6.4*) of which *Pax6.4* was lost before the diversification of vertebrates. The remaining three *Pax6* loci have experienced independent gene losses in different lineages. The mammalian and avian lineages have retained only one *Pax6* gene (*Pax6.1*), whereas the paired-less *Pax6.2* gene is additionally present in the *Xenopus*, lizard and teleost genomes. The fish specific genome duplication (FSGD (3R)) resulted in duplicate copies of the three gene loci (*Pax6.1a*/*Pax6.1b*; *Pax6.2a*/*Pax6.2b*; and *Pax6.3a*/*Pax6.3b*). Zebrafish is the only teleost which has retained both *Pax6.1* duplicates (*Pax6.1a* and *Pax6.1b*), whereas only one *Pax6.1* copy has been retained in acanthopterygians. Within the *Pax6.2* clade, zebrafish has retained *Pax6.2a* (syntenic to the lizard and *Xenopus Pax6.2* loci) whereas medaka, stickleback and fugu have retained the reciprocal copy of *Pax6.2* (*Pax6.2b*). Acanthopterygians (stickleback, medaka and fugu) are the only contemporary species to have retained a copy of *Pax6.3*. Gene losses are depicted by crosses and greyed-out branches/labels. The three WGD events are highlighted with stars. Hs, *Homo sapiens* (human); Mm, *Mus musculus* (mouse); Gg, *Gallus gallus* (chicken); Ac, *Anolis carolinensis* (lizard); Xt, *Xenopus tropicalis* (frog); Dr, *Danio rerio* (zebrafish); Ol, *Oryzias latipes* (medaka); Tr, *Takifugu rubripes* (fugu); Cm, *Callorhinchus milii* (elephant shark).

Based on the phylogenetic relationships of the various *Pax6* genes in vertebrates and their conservation of synteny and *cis*-regulatory domains, we propose the following model to explain the evolution of the family of *Pax6* genes in jawed vertebrates (Figure 9, Figure S6). The 1R and 2R that occurred in the stem vertebrate lineage gave rise to four copies of *Pax6* (*Pax6.1* to *Pax6.4*), one of which (*Pax6.4*) was lost before the diversification of gnathostomes. Of the remaining three genes one evolved into the canonical *Pax6* (*Pax6.1*), as represented by the well-studied contemporary mammalian *Pax6* locus. The second gene lost its 5′ exons encoding the paired domain and evolved to encode a paired-less form of *Pax6* (*Pax6.2*) expressed in a highly restricted pattern in the retina. This gene was subsequently lost independently in the mammalian and chicken lineages. The third copy (*Pax6.3*) was lost independently in the elephant shark lineage and in the common ancestor of tetrapods but retained in the ray-finned fish lineage. The origin of the *Pax6* family at the 1R/2R is consistent with the paucity of CNEs between the *Pax6* member loci as the WGDs occurred before the rapid and large-scale appearance of *cis*-regulatory sequences at the base of the jawed vertebrate lineage [53], [74].

The stem ray-finned fish lineage that diverged from the common ancestor of the tetrapods contained three *Pax6* loci (*Pax6.1*, *Pax6.2* and *Pax6.3*). The 3R that occurred in this lineage before the teleost radiation resulted in duplicate copies of each of these gene loci (*Pax6.1a*/*Pax6.1b*; *Pax6.2a*/*Pax6.2b*; and *Pax6.3a*/*Pax6.3b*). Among teleosts, zebrafish retained both copies of canonical *Pax6.1* due to *cis*-regulatory subfunctionalization, while the acanthopterygian ancestor lost one of its *Pax6.1* duplicates, resulting in a single copy of the canonical *Pax6* (*Pax6.1a*) locus in medaka, stickleback and fugu. Of the duplicate copies of the paired-less form of *Pax6*, one copy (*Pax6.2a*) was lost in the acanthopterygian ancestor, while the reciprocal copy (*Pax6.2b*) was lost in the zebrafish lineage. This has resulted in zebrafish retaining the *Pax6.2a* copy while medaka, stickleback and fugu retaining *Pax6.2b*. In the case of the third *Pax6* gene (*Pax6.3*), one of the two duplicate copies was lost before the divergence of the zebrafish and acanthopterygian lineages, whereas the second copy was lost in the zebrafish lineage after the split from the common ancestor of the acanthopterygians. Thus, no copies of the *Pax6.3* locus remain in zebrafish, whereas medaka, stickleback and fugu contain only one copy of this gene.

At present this model only presents a rough schematic of the progression of the *Pax6* gene family through vertebrate evolution, but future availability of whole genome sequences for additional vertebrate species will allow a more detailed model to emerge. The lamprey and hagfish genome sequences in particular could provide useful information on the *Pax6* duplications at the 1R and 2R events. In light of the presumed presence of the NRE and E-200 elements in the ancestral, pre-WGD *Pax6* locus we made alignments with the amphioxus (*Branchiostoma floridae*) and *Ciona Pax6* loci, but no sequence conservation apart from the coding exons was found.

Our model highlights the fact that the many pleiotropic functions of the key developmental regulator *Pax6* are shared between different members of a family of genes and isoforms in various species, and may be helpful in further dissection of species-specific differences in development and morphological variation. It therefore remains of great interest to continue the analysis of the *cis*-regulatory landscapes around *Pax6* genes from multiple species to trace the time-frame of acquisition of their *cis*-regulatory hardwiring and link the presence and divergence of enhancer elements with differences in expression patterns and in anatomical and developmental features.

## Materials and Methods

### Ethics statement

All mouse experiments were approved by the University of Edinburgh ethical committee (TR-11-08) and performed under UK Home Office license number PPL 60/3785.

### Sequence analysis

Genomic sequences were collected from Ensembl release 65, December 2011 [79] for these species: Human GRCh37 assembly (February 2009), Zebrafish Zv9 (Apr 2010), Medaka HdrR (Oct 2005), Tetraodon TETRAODON 8.0 (Mar 2007), Stickleback BROAD S1 (Feb 2006), Fugu FUGU 4.0 (Jun 2005). Sequences were manipulated using programs from the EMBOSS package [80]. Where necessary, missing regions of interest were incorporated following Blast searches [81] using human or fish Pax6 sequences. Ensembl gene annotation and protein predictions were used, in some cases supplemented with manual annotation using Genewise [82] for gene prediction from genomic sequence, with human or fish Pax6 protein as template. Other sources of information (proteins, ESTs) were used to further refine gene predictions and search for small exons, including exon 5a and 5′ non-coding exons. Protein sequences were aligned with the CLUSTALW program [83] and edited and displayed using the Genedoc software www.psc.edu/biomed/genedoc [84].

Genomic sequences were aligned, annotated and displayed using the PipMaker tools (http://bio.cse.psu.edu/pipmaker/) [62]. Evolutionary sequence comparison of the elephant shark *Pax6* loci with other species was prepared using the ‘glocal’ alignment program SLAGAN [85] with a window size of 100 bp and a minimal sequence identity of 70% and visualized using VISTA [86].

A neighbour-joining (NJ) tree for the Pax6 protein alignment was generated using MEGA (version 5.0.5; http://www.megasoftware.net/) [87]. A Poisson substitution model was used for distance calculation and 1000 bootstrap replicates were used for node support. The tree was viewed and edited using TreeView ver. 1.6.6 [88].

### Identification of elephant shark BACs

The DNA prepared from 92,160 clones of an elephant shark BAC library (IMCB_Eshark BAC library; unpublished) were pooled in three dimensions and used for identifying BAC clones by three-step PCR screening. The 1.4× coverage sequence of the elephant shark genome [66] was searched for the *Pax6* gene by BLAST. The search identified two scaffolds each containing a different *Pax6* fragment. PCR primers were designed for these fragments and used to identify positive BAC clones (23H6 and 37E6). These BACs were sequenced completely. BACs overlapping these seed-BACs were identified by PCR screening and sequenced completely. Altogether, four BACs were sequenced to obtain the sequence of *Pax6.1* locus (200B18, 50G15, 23H6 and 108J23) (GenBank JX135563) while two BACs were sequenced for the *Pax6.2* locus (87E22 and 37E6) (GenBank JX135564).

### Sequencing and assembly of BACs

BAC clones were sequenced using the standard shotgun sequencing method and gaps were filled by PCR amplification and primer walking. Sequencing was done using the BigDye Terminator Cycle Sequencing Kit (Applied Biosystems, USA). Sequences were processed and assembled using Phred-Phrap [89] and Consed [90]. Repetitive sequences were identified and masked using CENSOR at default settings [91]. Protein-coding genes were predicted using a combination of *ab initio* (e.g. FGENESH) and homology-based methods. Sequences for the human, chicken, lizard, *Xenopus* and zebrafish *Pax6* loci and genes were extracted from the UCSC Browser (http://genome.ucsc.edu/).

### RT–PCR and 5′RACE analyses of elephant shark *Pax6* genes

RT–PCR was performed using primers for *Pax6.1* and *Pax6.2* exons designed to encompass at least one intron. cDNA from 13 different tissues of elephant shark (brain, eye, gills, heart, kidney, liver, muscle, ovary, pancreas, spleen, intestine, testis and uterus) were used as template. Actin was amplified as a control to check for the quality of cDNA. The cycling conditions were 95°C for 2 mins, followed by 35 cycles of 95°C for 30 secs, 60°C for 30 secs and 72°C for 2 mins. 5′RACE was performed using the SMART cDNA amplification kit (BD Clontech, USA) and Advantage 2 PCR enzyme mix (BD Clontech, USA). The RACE products were sequenced either directly or after cloning into modified pBluescript.

### Cloning of conserved non-coding elements and genomic regions

CNEs selected for analysis in transgenic reporter assays were cloned by PCR amplification of the fragment containing the CNE plus flanking sequence from genomic DNA using Phusion high-fidelity polymerase (NEB). attB4 and attB1r sequences were included in the PCR primers for use with the Gateway recombination cloning system (Invitrogen). For zebrafish transgenic studies the amplified fragment was first cloned into the Gateway pP4P1r entry vector and sequenced using M13 forward and reverse primers for verification. Test elements in the pP4P1r vector were combined with a pDONR221 construct containing either a gata2 promoter-eGFP-polyA or a gata2 promoter-mCherry-polyA cassette, and recombined into a destination vector with a Gateway R4-R2 cassette flanked by Tol2 recombination sites [59], [92], [93].

For mouse transgenic LacZ reporter studies the selected fragments were PCR amplified using primers containing *Not*I and *Sal*I restriction sites and cloned into pGEM-T Easy vector (Promega). Fragments were then transferred into the hsp68-LacZ containing p610+ vector using the *Not*I and *Sal*I sites. For the elephant shark 6.2 NRE fragment the selected regions were amplified using primers containing Gateway compatible attB4 and attB1r sites, and cloned directly into an hsp68-LacZ vector containing a P4-P1r entry cassette. The forward primer also included an *Asc*I site to allow subsequent micro-injection fragment isolation.

The following primers were used:

Medaka pax6.1 intron 7: 5′-GCCCAACCAAGGTGAGCCTC-3′ and 5′-TTGGCAGCCATCTGAAGGTG

Medaka pax6.3 intron 7: 5′-GCTGGGACCACACTCTCCTCCA-3′ and 5′-TGGAGTTCACCGAGATGCCGT-3′

Zebrafish pax6.1a intron7: 5′-CCAATCAAGGTATGGCTGT-3′ and 5′-TGACTGTTGGCAACCATCTGA-3′

Zebrafish pax6.1b intron7: 5′-CCAAATCAAGGTGAGACAGCCA-3′ and 5′-TCCTGTTGCTGGCAACCGTCT-3′

Zebrafish pax6.1a 7CE1: 5′-CCAATCAAGGTATGGCTGT-3′ and 5′-GTTATGCCCTAAATCAAAGGCGT-3′

Zebrafish pax6.1a 7CE2: 5′-GTCCATAGGCTGTTAGTTTGGGT-3′ and 5′-ACTGCACGTATTTCCCCCTAG-3′

Zebrafish pax6.1a 7CE3: 5′-GCCACTGGTGTCTAACAAC-3′ and 5′-TTACAGCACTTTTCAGGCC-3′

Medaka pax6.1 7CE2: 5′-TGGCTCCAATCCCCGTTATAGGA-3′ and 5′-AAGGATGCCGCATGTGAGGGT-3′

Elephant shark pax6.1 7CE2: 5′-CTTCGACCATCAGCTGACAG-3′ and 5′-TCGGTCACTTTATGCCCACA-3′

Medaka pax6.3 7CE: 5′-GAAATGTGACAGCTGACAGGGT-3′ and 5′-CTTCATTTGGGGACTGAACAG-3′

Cm6.2NRE into p610+ and Tol2:

Cm6.2NRE forw: 5′-attB4-gcctcgcttcaaggccgatct-3′

Cm6.2NRE rev: 5′-attB1r-gccgtgcgggataaggtaga

Cm6.1 NRE into Tol2:

Cm6.1NRE forw: 5′-attB4-gctagaaccttcgcatctg

Cm6.2NRE rev: 5′-attB1r-gcatggcaaggctgcatgc

Zebrafish pax6.2_CNE1 into Tol2:

Drpax6.2_CNE1 forw: 5′-attB4-CCCTCATCCTCCCTCACATCT

Drpax6.2_CNE1 rev_short:5′-attB1r-TGTGACGTTGCGAGTGCGT

Drpax6.2_CNE1 rev_long:5′-attB1r-GCTCATACTGCGGTCCCAGA

### Transgenic reporter mice

Production of transgenic mice by micro-injection into mouse oocytes was performed according to standard procedures. For analysis, embryos were collected at the appropriate stages, washed in PBS and fixed for 1 hour in a solution of 1% formaldehyde; 0.2% glutaraldehyde; 2 mM MgCl_2_; 5 mM EGTA and 0.02% NP-40 in PBS. After fixation the embryos were washed in PBS containing 0.02% NP-40, before being stained for several hours at 37°C in the dark in a solution containing 5 mM K_3_Fe(CN)_6_; 5 mM K_4_Fe(CN)_6_.3H_2_O; 2 mM MgCl_2_; 0.01% sodium deoxycholate; 0.02% NP-40 and 0.1% 5-bromo-4-chloro-3-indolyl-β-D-galactopyranoside (X-gal). Embryos were photographed on a Leica MZ FLIII Microscope fitted with a Hamamatsu Orca-ER digital camera and a CRI micro-color filter. Table S1 contains details of the number of independent transgenic lines analysed and the observed sites of reporter gene expression.

### Comparison of *Pax6* synteny regions

Mapping of adjacent genes onto *Pax6* synteny regions was done using the Ensembl and UCSC genome browsers, with additional information obtained using the Genomicus website v66.01 [79] (http://www.dyogen.ens.fr/genomicus-66.01/cgi-bin/search.pl).

### rtPCR and RNA *in situ* analysis

Zebrafish *Pax6.2* in Ensembl: zgc193504; Ensembl identifier: ENSDARG00000053364; Location chromosome 3: 32,694,591–32,702,244.

RNA was isolated from pooled wild type zebrafish embryos from 1 dpf to 5 dpf using Tri-Reagent (Sigma). rtPCR was performed using Superscript III. PCR primers generating a fragment spanning several introns were designed for *Pax6*.2 and *Isl1*.

rtPCR primers for zebrafish *Pax6.2*:

forw: 5′-CTCAATGCACAGTCGGAGTG-3′

rev: 5′-TGCTGATTGAAGCTCTGCTGGT-3′

rtPCR primers for zebrafish Isl1:

islet1_fw CGGCGCACATATTCACATAC

islet1_rv TAAGCTTTAATACGACTCACTATAGGGAGAACGGACACGAACACATGAAA

### mRNA *in situ* hybridization

RNA *in situ* hybridization on fish embryos was performed as previously described [94].

The medaka *Pax6.1* RNA *in situ* probe was generated from the cDNA clone AJ000938 [63]; The RNA *in situ* probe for medaka *Pax6.3* was generated from EST clone MF01SSA182E11 (Genbank BJ013007 and BJ027294).

The RNA *in situ* probe for zebrafish *Pax6.2* was generated from the cDNA with primers:

T7: 5′-TAATACGACTCACTATAGGTGCTGATTGAAGCTCTGCTGGT-3′

5′-CTCAATGCACAGTCGGAGTG-3′

### Morpholino knock-down of zebrafish *Pax6.2*

A zebrafish *Pax6.2* antisense morpholino oligonucleotide was obtained from Gene Tools, LLC, with the following sequence: 5′ GTCATACATCAAATGTCACCTGTGA 3′, directed against the translation start site of the gene.

As control we used the Gene ToolsLLC standard negative control morpholino: 5′ CCTCTTACCTCAGTTACAATTTATA 3′. The morpholinos were injected into 1 to 2-cell stage of at least 100 embryos to deliver an approximate amount of 2.5 ng per embryo.

### Generation of zebrafish transgenic lines

Zebrafish were maintained in a recirculating water system according to standard protocol [95]. Embryos were obtained by breeding adult fish of standard stains (AB and WIK) and raised at 28.5°C as described [95]. Embryos were staged by hours post fertilization (hpf) as described [96].

Reporter plasmids were isolated using Qiagen miniprep columns and were given extra purification via a Qiagen PCR purification column (Qiagen), and diluted to 50 ng/microliter with DNAse/RNAse free water. *tol2* transposase RNA was synthesized from a *NotI*-linearized *pCS2-TP* plasmid [93] using the SP6 mMessage mMachine kit (Ambion), and similarly diluted to 50 ng/microliter. Equal volumes of the reporter construct(s) and the transposase RNA were mixed immediately prior to injections to give an injection solution containing 25 ng/microliter of DNA and 25 ng/microliter of transposase RNA. 1–2 nl of the solution was micro-injected per embryo into the cytoplasm of 200 embryos at the 1- to 2-cell stage. Embryos were screened for mosaic fluorescence at 1–5 days post-fertilization (dpf) and raised to adulthood. Germline transmission was identified by mating of sexually mature adults to wild-type fish and examining their progeny for fluorescence. Positive embryos were raised to adulthood and lines were maintained by outcrossing. Fish showing the best representative expression pattern for each construct were selected for confocal imaging. A summary table detailing numbers of independent lines analysed for each construct and their expression sites is included in Table S1.

### Imaging of zebrafish reporter transgenic embryos

Embryos for imaging were treated with 0.003% PTU (1-phenyl 2-thio-urea) from 24 hpf to prevent pigmentation. Embryos selected for imaging were anaesthetised with tricaine and mounted in 1% low-melting agarose. Images were taken on a Zeiss LSM510 confocal microscope and processed using Fiji software.

## Supporting Information

Figure S1 PIP plot using the stickleback *pax6.3* locus as baseline sequence. The *pax6.3* loci contain several CNEs that are specifically conserved between acanthopterygian fish (arrowheads). There are no CNEs conserved between the loci of *pax6.3* and *pax6.1*, with the exception of the E-200 element (red box). The position of the intron 7 conserved element (7CE(6.3)) is indicated by a blue box. *Dkk3*, dickkopf 3 homolog, *Dnajc24*, dna J homolog, subfamily C.
(TIF)

Figure S2 Protein alignment of representative members of the vertebrate *Pax6* gene family. The alignment highlights the separation into three branches representing the *pax6.1*, *pax6.2* and *pax6.3* clades. *Pax6.1* is the canonical *Pax6* gene found in all vertebrates. *Pax6.2* encodes a paired-less form of *Pax6*. The *pax6.3* gene lacks an alternative exon 5a and thus does not have the ability to encode the *Pax6*(5a) isoform. The position of exon 5a is shown. The paired domain is indicated by a red line above the sequence. A blue line highlights the homeodomain and a black line shows the PST-rich transactivation domain. H.sap, *Homo sapiens* (human); A.car, *Anolis carolinensis* (lizard); X.tro, *Xenopus tropicalis* (frog); L.cha, *Latimeria chalumnii* (coelacanth); C.mil, *Callorhinchus milii* (elephant shark); Ol, *Oryzias latipes* (medaka); G.acu, *Gasterosteus aculeatus* (stickleback); T.rub, *Takifugu rubripes* (fugu); T.nig, Tetraodon nigroviridis (tetraodon); D.rer, *Danio rerio* (zebrafish).
(TIF)

Figure S3 Multispecies alignment of the E-200 element. The novel E-200 long-range enhancer is located approximately 215 kb upstream of human *PAX6*. Strong sequence conservation is found between the E-200 elements of *Pax6.1* loci over a 400 bp region. Conservation with the *pax6.3* loci of Tetraodon and stickleback is limited to the central core of the element. Hsap, *Homo sapiens* (human); Mmus, *Mus musculus* (mouse); Ggal, *Gallus gallus* (chicken); Xt, *Xenopus tropicalis* (frog); Drer, *Danio rerio* (zebrafish); Gacu, *Gasterosteus aculeatus* (stickleback); Olat, *Oryzias latipes* (medaka); Trub, *Takifugu rubripes* (fugu).
(TIF)

Figure S4 Phylogeny and synteny relationships between the vertebrate*Pax6* loci (extended version of Figure 7 including more species). Phylogeny/ontogeny of the *Pax6* gene family is supported by comparison of gene content in the synteny regions of the genes. A) The *Pax6.1* gene resides in an ancient synteny block that is perfectly conserved from elephant shark to human. Gene content is also largely conserved in the *Pax6.1* upstream region in teleost fish, while synteny breaks beyond the *Immp1l* gene. The duplicate zebrafish *Pax6.1* loci show a clear sub-partitioning of the genes in the syntenic region. B) Pax6.3 is only found in acanthopterygian fish and forms a mini block of conserved synteny with the *dnajc24* gene. C) The paired-less *Pax6.2* gene is found in a region of conserved synteny with the *Rcn3* and *Nosip* genes in the elephant shark, *Xenopus* and lizard genomes. In mammals there is no *pax6* adjacent to *Rcn3* and *Nosip*. Reciprocal *pax6.2* duplicates have been retained in teleosts. Zebrafish *pax6.2a* is found in synteny with *rcn3* and *nosip*, but the *rcn3*/*nosip* synteny region in medaka does not contain *pax6*. Instead a *pax6.2* (*pax6.2b*) is found between *prss22* and *mmp25* in acanthopterygians, where conversely it is absent from this region in zebrafish. *Rcn1*, reticulocalbin 1, *Elp4*, elongator protein subunit 4, *Wt1*, Wilms tumour 1, *Immp1l*, inner mitochondrial membrane peptidase like 1, *Dnajc24*, dna J homolog, subfamily C, member 24, *Mmped2*, metallophosphoesterase domain containing 2, *Rcn3*, reticulocalbin 3, *Nosip*, nitric oxide synthase interacting protein, *Fcgrt*, Fc fragment of IgG, receptor, transporter, alpha, *Prss22*, protease serine 22, *Prr12*, proline rich 12, *Irf3*, interferon regulatory factor 3; *Mmp25*, matrix metallo peptidase 25, *Trpm4*, transient receptor potential cation channel, subfamily M member 4, *Ca4*, carbonic anhydrase IV; *Mapk7*, mitogen activated protein kinase 7, *Ric8a*, resistance to inhibitors of cholinesterase 8 homolog A.
(TIF)

Figure S5 Depletion of *Pax6.2* by morpholino knock-down. A) a *Pax6.2* morpholino. and B) a control morpholino were Injected into zebrafish oocytes. Pools of embryos were fixed at 2 dpf and tested for the presence of *Pax6.2* transcript by *in situ* hybridization. *Pax6.2* ISH signal was absent or greatly reduced in the majority of pax6.2 morpholino injected embryos, but was unaffected by control morpholino injections. C, D) Diameter of the eye was measured relative to the total body size of the embryos, and the average ratio of eye diameter versus body length was calculated. E) Chart showing the eye diameter of pax6.2 morphants was on average 30% reduced compared to embryos injected with a non-specific morpholino.
(TIF)

Figure S6 Schematic reconstruction of the ontology of the *Pax6* gene family in vertebrate evolution. Three clades of *Pax6* orthologs remain in various compositions in vertebrate genomes to date: the canonical *Pax6.1* loci, the paired-less *Pax6.2* loci and the acanthopterygian *Pax6.3* loci lacking the alternative exon5a. The duplicate *Pax6* loci are proposed to have arisen during the 2 rounds of whole genome duplication (WGD) in early vertebrate evolution, while the zebrafish pax6.1a and pax6.1b duplicates and the reciprocal copies of pax6.2 (zebrafish pax6.2a and acanthopterygian pax6.2b) result from the fish specific genome duplication (FSGD). The time line of vertebrate evolution runs from the dawn of the vertebrate lineage on the left to the present on the right, but is not to scale.
(TIF)

Table S1 Overview of the constructs used to evaluate cis-regulatory activity of conserved non-coding elements. For each construct the species of origin and the transgenic model system used are given, followed by the number of stable transgenic lines analysed and the observed sites of reporter expression.
(DOC)

Video S1 Optical Projection Tomography (OPT) movie of pax6.2 expression. The OPT movie demonstrates the highly specific expression of *pax6.2* in the retina. 2 dpf zebrafish embryos were *in situ* hybridized with a *pax6.2* antisense probe. A representative embryo was scanned by OPT and an AVI movie was created using Drishti software.
(AVI)
