# Supplementary figures and images for: Sequencing of Pax6 Loci from the Elephant Shark Reveals a Family of Pax6 Genes in Vertebrate Genomes, Forged by Ancient Duplications and Divergences
Source: PLoS Genet. 2013 Jan 24;9(1):e1003177. doi: 10.1371/journal.pgen.1003177 (PMC3554528; doi:10.1371/journal.pgen.1003177)

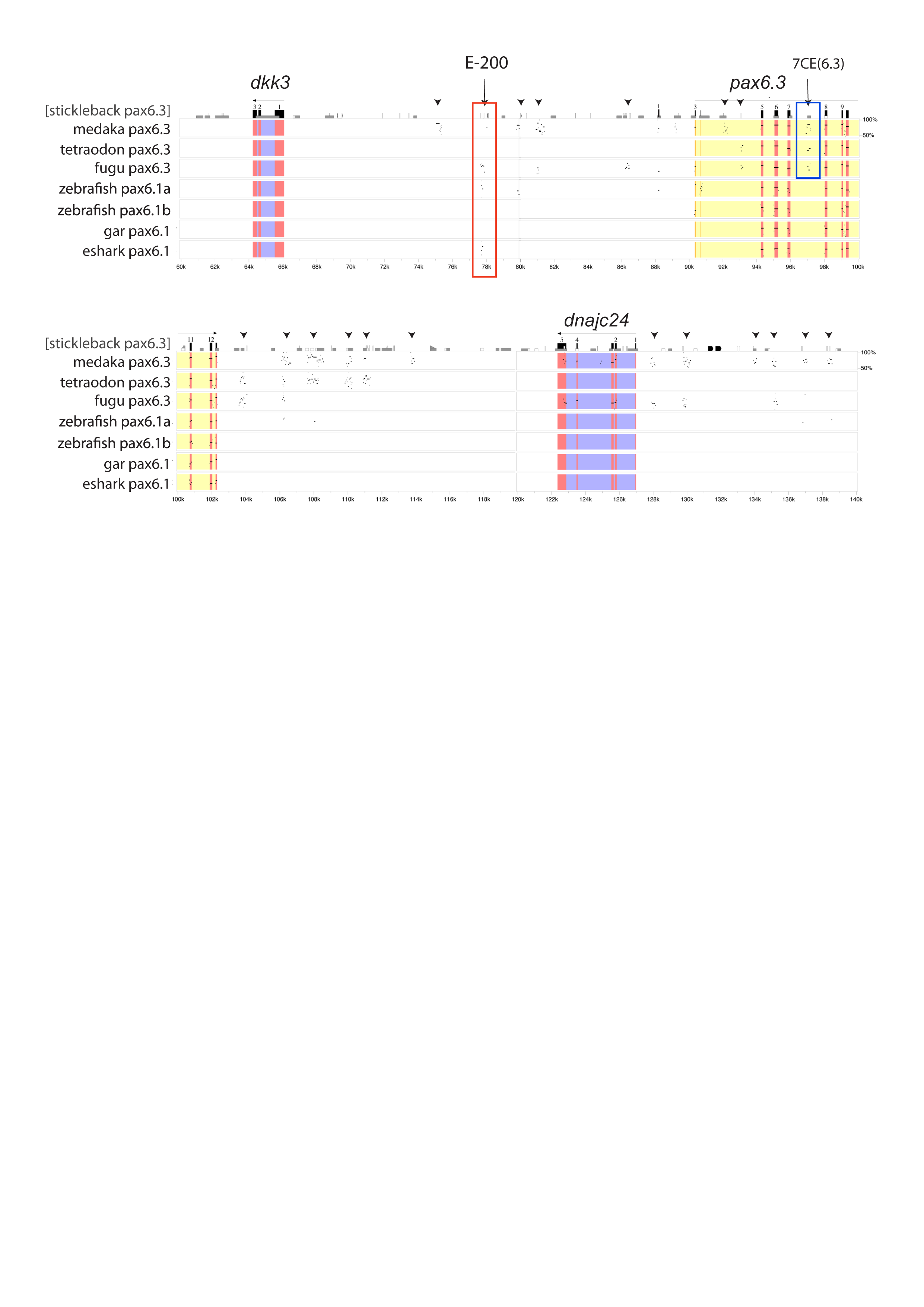

Supplement: Figure S1 — PIP plot using the stickleback pax6.3 locus as baseline sequence. The pax6.3 loci contain several CNEs that are specifically conserved between acanthopterygian fish (arrowheads). There are no CNEs conserved between the loci of pax6.3 and pax6.1, with the exception of the E-200 element (red box). The position of the intron 7 conserved element (7CE(6.3)) is indicated by a blue box. Dkk3, dickkopf 3 homolog, Dnajc24, dna J homolog, subfamily C. (TIF) [file pgen.1003177.s001.tif]

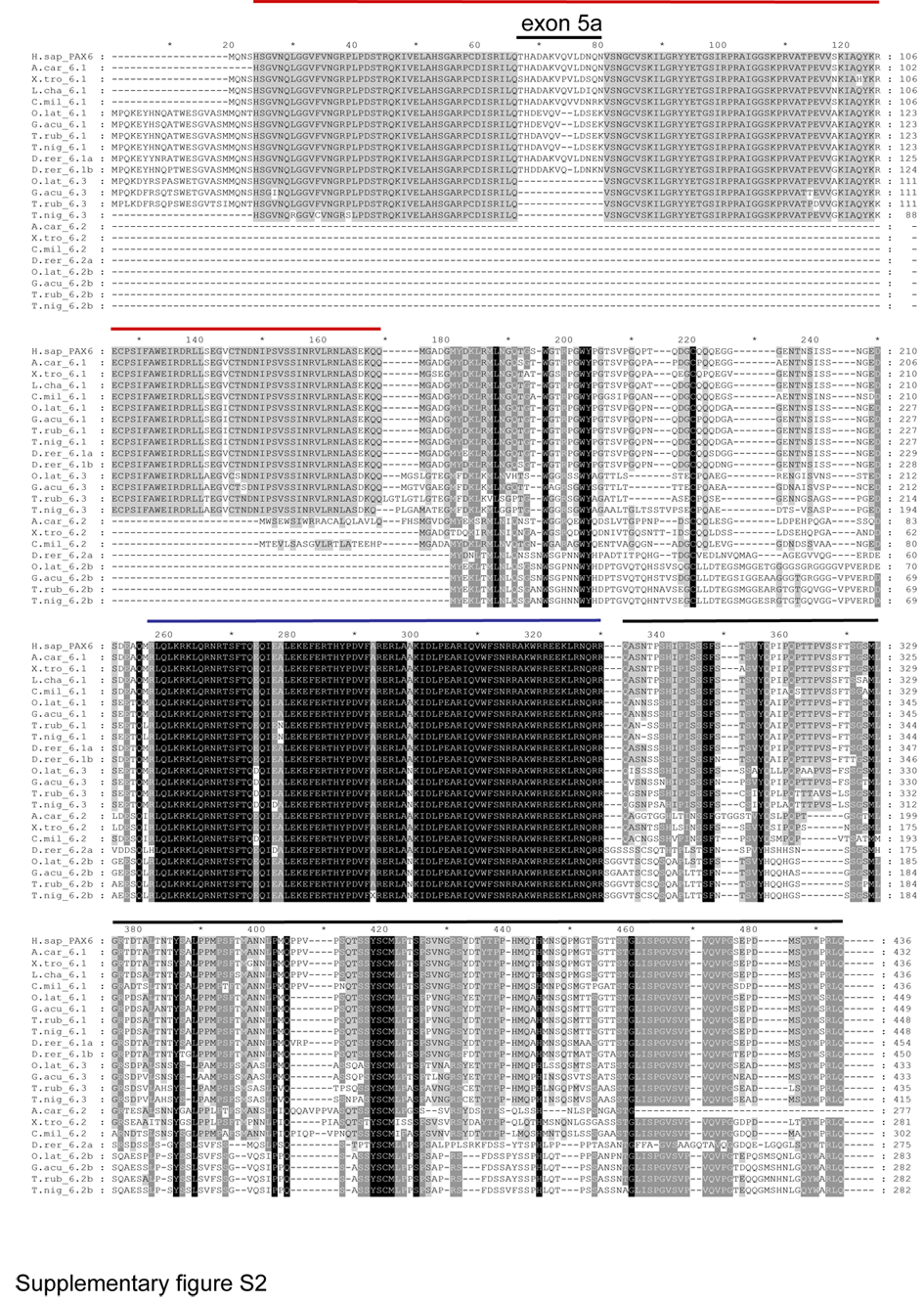

Supplement: Figure S2 — Protein alignment of representative members of the vertebrate Pax6 gene family. The alignment highlights the separation into three branches representing the pax6.1, pax6.2 and pax6.3 clades. Pax6.1 is the canonical Pax6 gene found in all vertebrates. Pax6.2 encodes a paired-less form of Pax6. The pax6.3 gene lacks an alternative exon 5a and thus does not have the ability to encode the Pax6(5a) isoform. The position of exon 5a is shown. The paired domain is indicated by a red line above the sequence. A blue line highlights the homeodomain and a black line shows the PST-rich transactivation domain. H.sap, Homo sapiens (human); A.car, Anolis carolinensis (lizard); X.tro, Xenopus tropicalis (frog); L.cha, Latimeria chalumnii (coelacanth); C.mil, Callorhinchus milii (elephant shark); Ol, Oryzias latipes (medaka); G.acu, Gasterosteus aculeatus (stickleback); T.rub, Takifugu rubripes (fugu); T.nig, Tetraodon nigroviridis (tetraodon); D.rer, Danio rerio (zebrafish). (TIF) [file pgen.1003177.s002.tif]

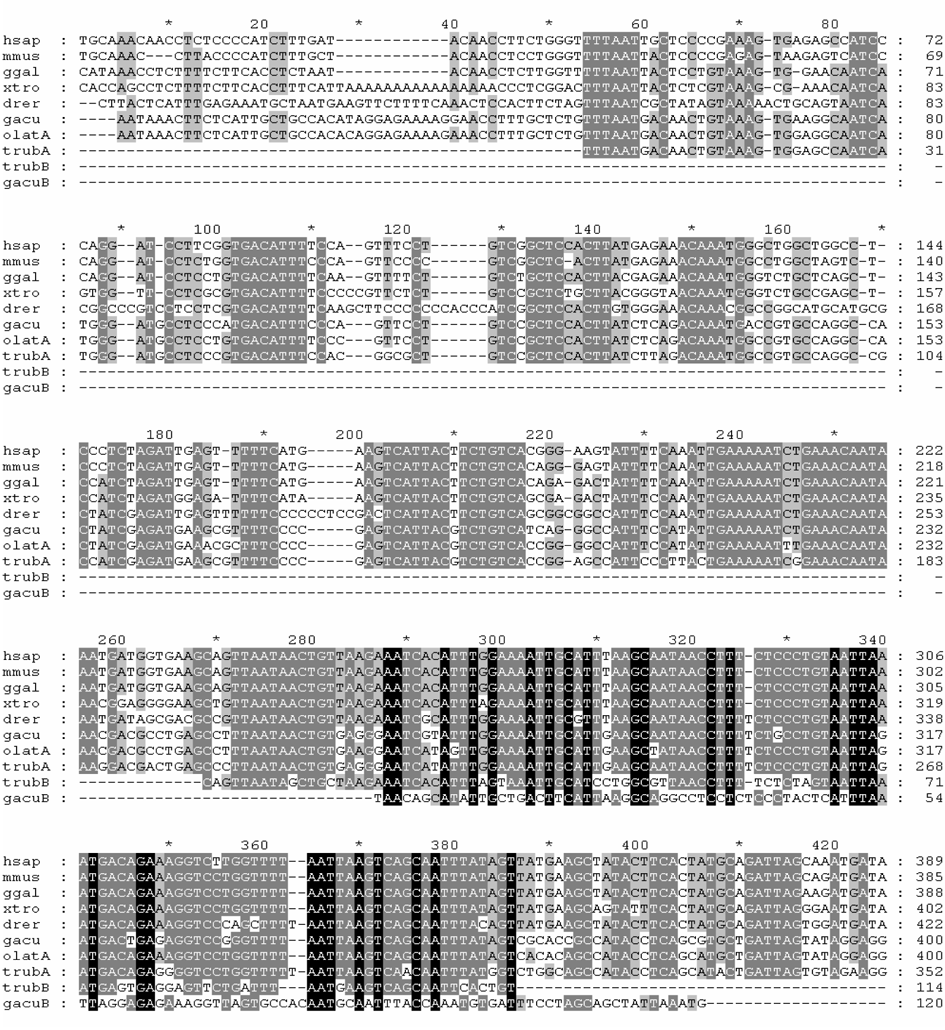

Supplement: Figure S3 — Multispecies alignment of the E-200 element. The novel E-200 long-range enhancer is located approximately 215 kb upstream of human PAX6. Strong sequence conservation is found between the E-200 elements of Pax6.1 loci over a 400 bp region. Conservation with the pax6.3 loci of Tetraodon and stickleback is limited to the central core of the element. Hsap, Homo sapiens (human); Mmus, Mus musculus (mouse); Ggal, Gallus gallus (chicken); Xt, Xenopus tropicalis (frog); Drer, Danio rerio (zebrafish); Gacu, Gasterosteus aculeatus (stickleback); Olat, Oryzias latipes (medaka); Trub, Takifugu rubripes (fugu). (TIF) [file pgen.1003177.s003.tif]

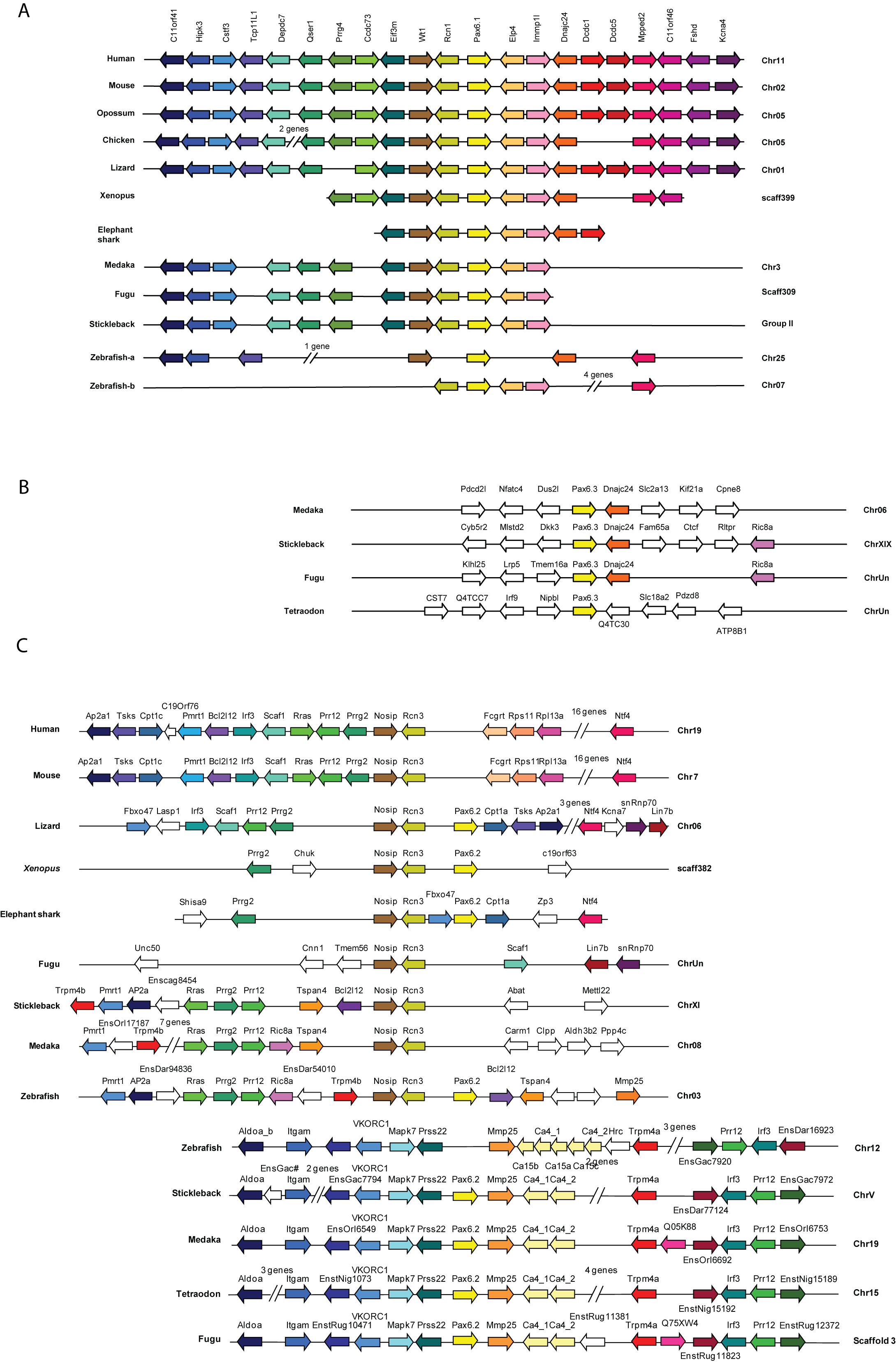

Supplement: Figure S4 — Phylogeny and synteny relationships between the vertebratePax6 loci (extended version of Figure 7 including more species). Phylogeny/ontogeny of the Pax6 gene family is supported by comparison of gene content in the synteny regions of the genes. A) The Pax6.1 gene resides in an ancient synteny block that is perfectly conserved from elephant shark to human. Gene content is also largely conserved in the Pax6.1 upstream region in teleost fish, while synteny breaks beyond the Immp1l gene. The duplicate zebrafish Pax6.1 loci show a clear sub-partitioning of the genes in the syntenic region. B) Pax6.3 is only found in acanthopterygian fish and forms a mini block of conserved synteny with the dnajc24 gene. C) The paired-less Pax6.2 gene is found in a region of conserved synteny with the Rcn3 and Nosip genes in the elephant shark, Xenopus and lizard genomes. In mammals there is no pax6 adjacent to Rcn3 and Nosip. Reciprocal pax6.2 duplicates have been retained in teleosts. Zebrafish pax6.2a is found in synteny with rcn3 and nosip, but the rcn3/nosip synteny region in medaka does not contain pax6. Instead a pax6.2 (pax6.2b) is found between prss22 and mmp25 in acanthopterygians, where conversely it is absent from this region in zebrafish. Rcn1, reticulocalbin 1, Elp4, elongator protein subunit 4, Wt1, Wilms tumour 1, Immp1l, inner mitochondrial membrane peptidase like 1, Dnajc24, dna J homolog, subfamily C, member 24, Mmped2, metallophosphoesterase domain containing 2, Rcn3, reticulocalbin 3, Nosip, nitric oxide synthase interacting protein, Fcgrt, Fc fragment of IgG, receptor, transporter, alpha, Prss22, protease serine 22, Prr12, proline rich 12, Irf3, interferon regulatory factor 3; Mmp25, matrix metallo peptidase 25, Trpm4, transient receptor potential cation channel, subfamily M member 4, Ca4, carbonic anhydrase IV; Mapk7, mitogen activated protein kinase 7, Ric8a, resistance to inhibitors of cholinesterase 8 homolog A. (TIF) [file pgen.1003177.s004.tif]

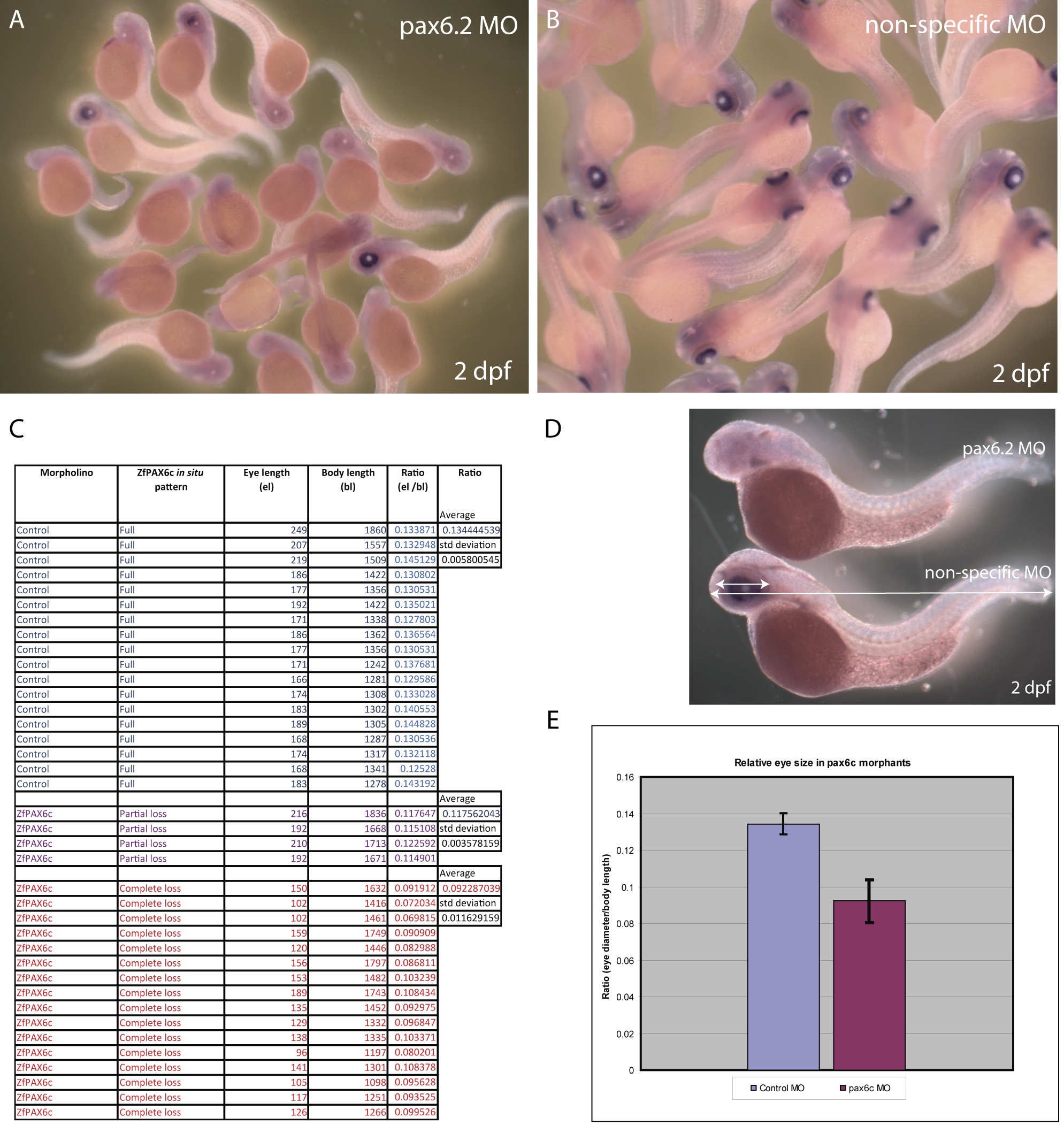

Supplement: Figure S5 — Depletion of Pax6.2 by morpholino knock-down. A) a Pax6.2 morpholino. and B) a control morpholino were Injected into zebrafish oocytes. Pools of embryos were fixed at 2 dpf and tested for the presence of Pax6.2 transcript by in situ hybridization. Pax6.2 ISH signal was absent or greatly reduced in the majority of pax6.2 morpholino injected embryos, but was unaffected by control morpholino injections. C, D) Diameter of the eye was measured relative to the total body size of the embryos, and the average ratio of eye diameter versus body length was calculated. E) Chart showing the eye diameter of pax6.2 morphants was on average 30% reduced compared to embryos injected with a non-specific morpholino. (TIF) [file pgen.1003177.s005.tif]

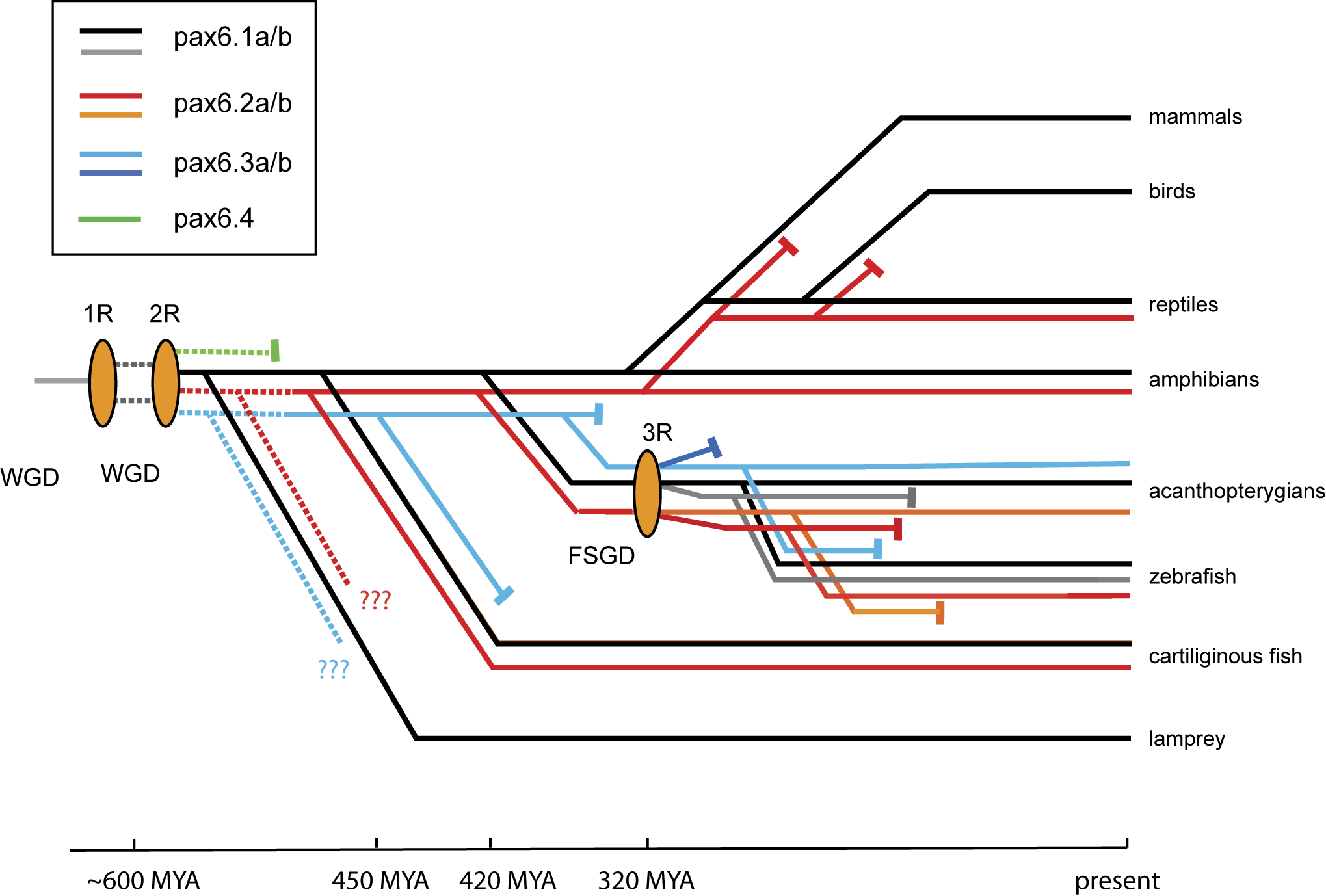

Supplement: Figure S6 — Schematic reconstruction of the ontology of the Pax6 gene family in vertebrate evolution. Three clades of Pax6 orthologs remain in various compositions in vertebrate genomes to date: the canonical Pax6.1 loci, the paired-less Pax6.2 loci and the acanthopterygian Pax6.3 loci lacking the alternative exon5a. The duplicate Pax6 loci are proposed to have arisen during the 2 rounds of whole genome duplication (WGD) in early vertebrate evolution, while the zebrafish pax6.1a and pax6.1b duplicates and the reciprocal copies of pax6.2 (zebrafish pax6.2a and acanthopterygian pax6.2b) result from the fish specific genome duplication (FSGD). The time line of vertebrate evolution runs from the dawn of the vertebrate lineage on the left to the present on the right, but is not to scale. (TIF) [file pgen.1003177.s006.tif]
