# Supplementary material for: Sequencing of Pax6 Loci from the Elephant Shark Reveals a Family of Pax6 Genes in Vertebrate Genomes, Forged by Ancient Duplications and Divergences
Source: PLoS Genet. 2013 Jan 24;9(1):e1003177. doi: 10.1371/journal.pgen.1003177 (PMC3554528; doi:10.1371/journal.pgen.1003177)
Supplement: Table S1 — Overview of the constructs used to evaluate cis-regulatory activity of conserved non-coding elements. For each construct the species of origin and the transgenic model system used are given, followed by the number of stable transgenic lines analysed and the observed sites of reporter expression. (DOC) [file pgen.1003177.s007.doc]

| S.No. | Element name | Origin | Model organism for reporter transgenic assay | Total number of stable transgenic lines analysed | Sites of reporter expression driven by the element |
| --- | --- | --- | --- | --- | --- |
| 1 | Dr_6.1a_7CE2 | *Danio rerio* | *Danio rerio* | 4 | Diencephalon (4/4)  Heart (1/4) |
| 2 | Dr_6.1a_7CE3 | *Danio rerio* | *Danio rerio* | 4 | Hindbrain (4/4)  Lens (1/4) |
| 3 | Dr_6.1a_int7 | *Danio rerio* | *Danio rerio* | 6 | Diencephalon (5/6)  Hindbrain (6/6)  Lens (1/6) |
| 4 | Dr_6.1b_int7 | *Danio rerio* | *Danio rerio* | 6 | Hindbrain (6/6)  Retina (2/6)  Fin buds (1/6) |
| 5 | Ol_6.1_int7 | *Oryzias latipes* | *Danio rerio* | 6 | Diencephalon (5/6)  Hindbrain (6/6)  Telencephalon (1/6) |
| 6 | Ol_6.3_int7 | *Oryzias latipes* | *Danio rerio* | 4 | Retina (4/4)  Lens (2/4)  Heart (1/4) |
| 7 | Ol_6.3_7CE | *Oryzias latipes* | *Danio rerio* | 5 | Retina (5/5)  Lens (2/5) |
| 8 | E-200 | *Homo sapiens* | *Danio rerio* | 3 | Olfactory bulbs (3/3)  Hindbrain (2/3)  Lens (1/3) |
| 9 | E-200 | *Homo sapiens* | *Mus musculus* | 4 | Olfactory bulbs (3/4)  Lateral olfactory tract (3/4)  Cerebellum (3/4)  Pontine nuclei (2/4)  Lens (1/4)  Genital ridge (1/4) |
| 10 | Esh61_NRE | *Callorhinchus milii* | *Danio rerio* | 4 | Retina (4/4)  Fin buds (1/4) |
| 11 | Esh62_NRE | *Callorhinchus milii* | *Danio rerio* | 4 | Retina (4/4)  Lateral line (1/4) |
| 12 | Esh62_NRE | *Callorhinchus milii* | *Mus musculus* | 4 | Retina (4/4)  Dorsal root ganglia (2/4)  Midbrain (1/4)  Telencephalon (1/4)  Branchial arches (1/4)  Neural tube (1/4)  Genital ridge (1/4)  Maxillary/mandible (1/4)  Limb buds (1/4) |
| 13 | CNE(6.2)long | *Danio rerio* | *Danio rerio* | 5 | Retina (5/5)  Telencephalon (1/5) |
| 14 | CNE(6.2)short | *Danio rerio* | *Danio rerio* | 3 | Retina (3/3) |

Table S1.
